# Single-cell immune profiling of blood and thrombus in cancer-associated thrombosis

**DOI:** 10.1016/j.isci.2026.117151

**Published:** 2026-08-20

**Authors:** Linlin Zhou, Yin Xia, Bingkun Chen, Xiaowen Lin, Hung-Chih Yang, Chen-Wei Yu

**Affiliations:** 1 Institute of Immunotherapy, Fujian Medical University, Fuzhou, China; 2 Department of Vascular Surgery, the Affiliated People’s Hospital of Fujian University of Traditional Chinese Medicine, Fuzhou, China; 3 The School of Basic Medical Sciences, Fujian Medical University, Fuzhou, China; 4 Department of Microbiology, National Taiwan University College of Medicine and Department of Internal Medicine, National Taiwan University Hospital, Taipei, Taiwan; 5 Department of Statistics and Information Science, Fu Jen Catholic University, New Taipei City, Taiwan

**Keywords:** deep vein thrombosis, cancer-associated thrombosis, single-cell immune profiling

## Abstract

Cancer-associated thrombosis (CAT) is a major cause of morbidity and mortality in patients with cancer, but the immune landscape of the thrombus itself is poorly mapped. We combined high-parameter mass cytometry of paired peripheral blood and venous thrombi from non-cancer deep vein thrombosis (DVT) and CAT with an integrated single-cell RNA-sequencing atlas to profile immune remodeling at single-cell resolution. Relative to non-cancer DVT, CAT thrombi showed expansion of CXCR3^+^CD11b^+^ myeloid subsets, loss of conventional CD4^+^ and CD8^+^ T cells, and accumulation of unconventional double-negative T cells, alongside systemic depletion of T-bet^+^EOMES^+^ natural killer (NK) cells. We also detected PD-L1 on the CD45^−^ non-immune compartment of CAT thrombi and found a positive association between hemoglobin and FOXP3^+^CD8^+^ T cells. These exploratory findings nominate chemokine- and checkpoint-related pathways for future study and provide an open single-cell resource for thrombo-immunology research.

## Highlights

• CAT thrombi show myeloid expansion and loss of conventional T cells versus DVT
• Suppressive PD-1^+^PD-L1^+^ and CXCR3^+^ myeloid subsets accumulate in cancer thrombi
• PD-L1 marks the CD45^−^ non-immune compartment of cancer-associated thrombi
• Hemoglobin tracks with FOXP3^+^CD8^+^ T cells across blood and thrombus

## Graphical abstract

**Figure:**
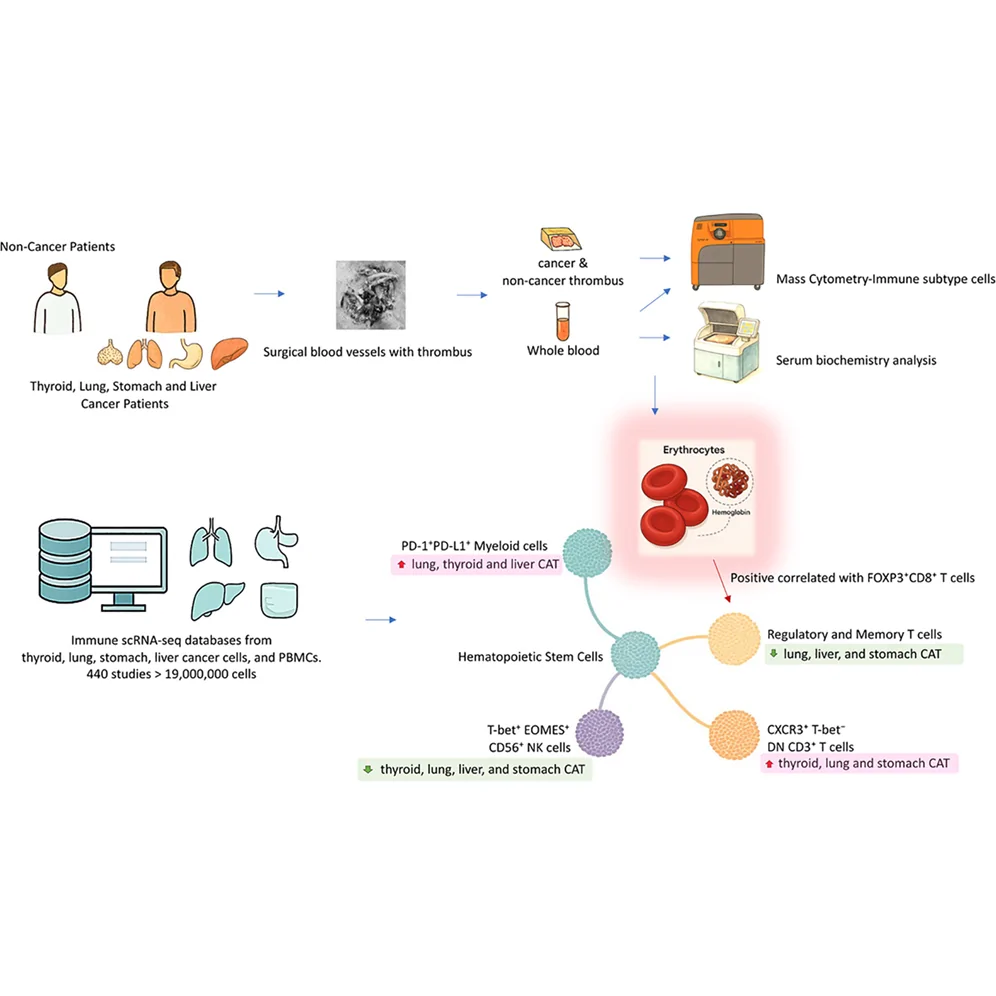

## Teaser

Immunology; Hematology; Cancer

## Introduction

Venous thromboembolism (VTE), comprising deep vein thrombosis (DVT) and pulmonary embolism (PE), arises from clot formation within the deep venous system. In patients with cancer, these events are collectively referred to as cancer-associated thrombosis (CAT). CAT is common and clinically serious, contributing substantially to morbidity and ranking among the leading causes of death in cancer after disease progression.^1^ Patients with CAT have a poorer prognosis than cancer patients without VTE, with higher all-cause mortality and increased risks of recurrent VTE and major bleeding.^2^ In cancer, abnormalities in Virchow’s triad are frequent: stasis of blood flow due to immobility or venous compression, endothelial injury from tumor invasion and anticancer therapies, and a hypercoagulable milieu driven by tumor-derived procoagulant factors and systemic inflammation. Together, these changes promote thrombosis in patients with cancer.^3^^,^^4^

Multiple, convergent mechanisms promote thromboembolic events in cancer. Risks vary by tumor type, reflecting cancer-specific biology. Cancer cells express procoagulants and release mediators that directly contribute to hypercoagulability.^5^^,^^6^ Furthermore, the immune system is crucial in developing and progressing venous thrombosis in patients with cancer.^7^ For instance, neutrophil extracellular traps provide a scaffold for fibrin and platelets, while inflammatory signaling and immune-endothelial interactions in the cancer microenvironment further potentiate thrombosis.^8^ In addition to neutrophils, monocytes recruited to the venous wall are a principal source of tissue factor (TF), initiating the extrinsic coagulation pathway and subsequent fibrin deposition.^9^ In the stenosis model of DVT, the ablation of TF within myeloid leukocytes effectively precludes thrombus formation while leaving leukocyte recruitment unaffected.^10^ Several studies have indicated that cancer cells promote venous thrombus formation via cancer cell-derived microvesicles, cell-free DNA, and damage-associated molecular patterns.^11^^,^^12^^,^^13^^,^^14^ The release of inflammatory cytokines and chemokines by both cancer cells and immune cells can promote a localized prothrombotic environment. Of note, DVT in patients with cancer is probably driven by combinations of different pathways rather than a single pathway. Importantly, CAT does not represent a single, cancer-type-specific entity in the way that targeted therapies have specific molecular indications. Rather, it reflects a shared pathophysiological state driven by common mechanisms including TF expression, neutrophil extracellular traps, plasminogen activator inhibitor-1, and inflammatory cytokine signaling, onto which cancer-type-specific modulators are superimposed (for example, podoplanin in brain tumors, the IL-6/thrombopoietin axis in ovarian cancer, and ALK rearrangements in non-small cell lung cancer). This conceptual framework motivates the present study design, in which the primary comparison contrasts CAT with non-cancer DVT to identify the shared CAT-associated immune signature, while cancer-type-specific observations are reported as descriptive, hypothesis-generating signals that may inform future targeted investigations.^15^^,^^16^

Immunotherapy is a major advance in oncology, yet comparative immune profiling of venous thrombi in non-cancer DVT versus CAT remains limited. Much of the prior immune work focuses on atherosclerotic arterial plaques; in contrast, the differences in circulating and intrathrombus immune compositions between non-CAT DVT and CAT are still unclear, with most studies emphasizing macrophage-driven plaque instability rather than venous thrombus biology.^17^^,^^18^ To address this gap, we profile the single-cell immune landscape of DVT and CAT thrombi and paired blood samples, delineating immune populations and inferring potential interactions within the thrombus microenvironment.

## Results

### Immune cell composition across blood, thrombi, primary tumor, and matched normal tissues

**Figure 1:**
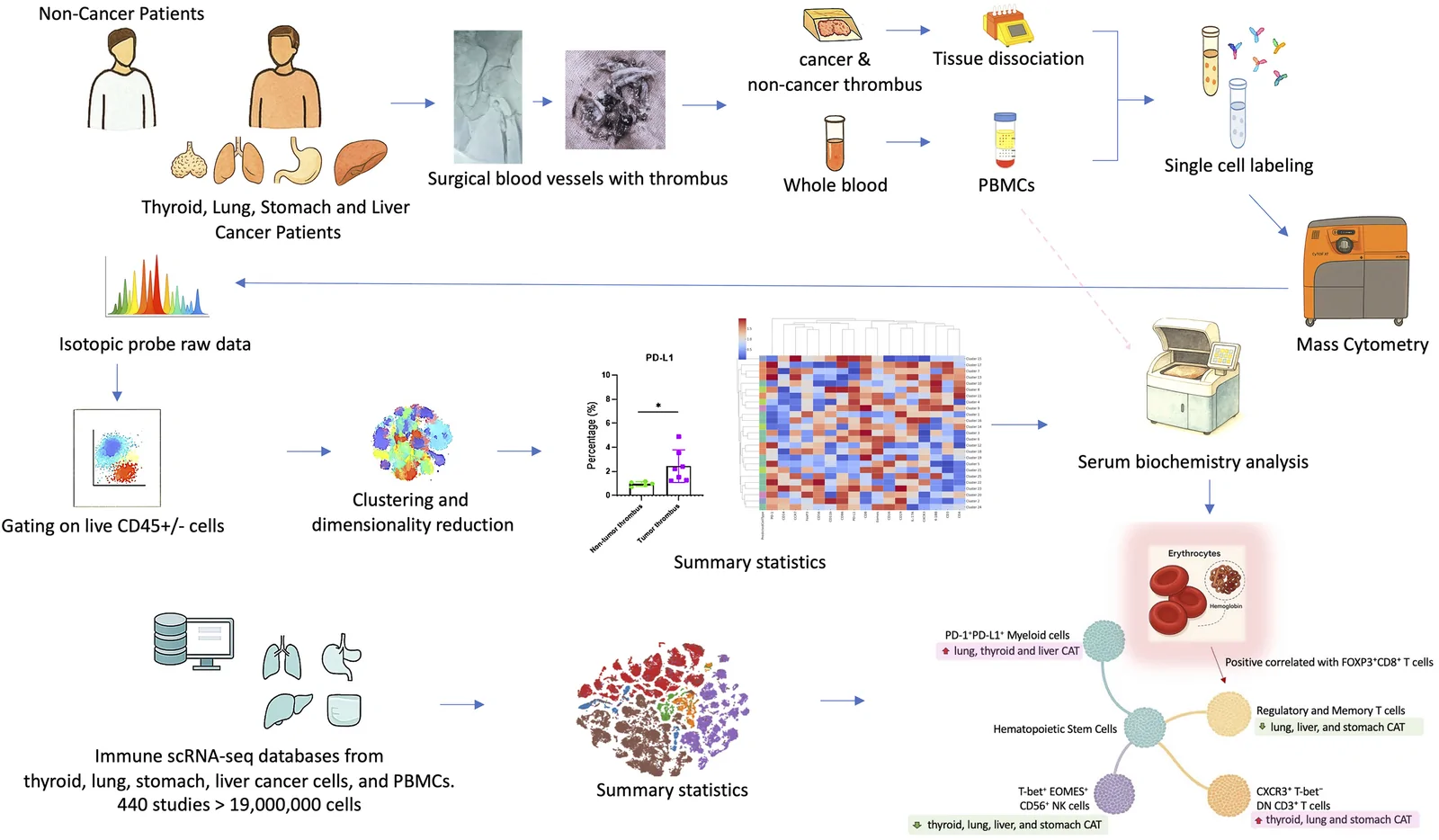
Integrated experimental and analytical pipeline for profiling immune landscapes in non-cancer DVT and CAT Individuals undergoing surgery for venous thrombosis were stratified into a non-cancer DVT cohort (*n* = 5) and a cancer cohort encompassing thyroid, lung, stomach, and liver malignancies (*n* = 7; treatment-naive at sampling). Thrombi were mechanically and enzymatically dissociated, and peripheral-blood mononuclear cells (PBMCs) were isolated by density-gradient centrifugation. All single-cell suspensions were barcoded, stained with a specific antibody panel, and profiled by mass cytometry (CyTOF). Live CD45^+^ and CD45^−^ cells were gated, followed by unsupervised clustering, dimensionality reduction (t-SNE/UMAP), and quantitative comparison of immune-cell frequencies and functional states. Routine hematology and biochemical indices were measured in parallel to enable immuno-clinical correlation analyses. Public scRNA-seq atlases of primary tumors and PBMCs were integrated to annotate CyTOF-defined populations and extend cross-tissue comparisons. Cell-subset enrichments and biomarker correlations were contrasted across compartments (PBMCs, non-cancer DVT thrombi, and CAT thrombi), revealing conserved and cancer-specific immune-remodeling signatures.

To characterize immune alterations associated with CAT, we generated mass cytometry (CyTOF) data on the peripheral-blood mononuclear cells (PBMCs) and venous thrombi from the 12 patients of our directly profiled cohort and integrated these primary patient-level findings with an external single-cell RNA sequencing (scRNA-seq) atlas drawn from the BioTuring Talk2Data v5 platform (Figure 1). All primary, patient-level findings reported in this study, including all DVT-versus-CAT comparisons, all per-patient compositional changes, and all clinical-correlation regressions, derive from the directly profiled CyTOF cohort. The integrated scRNA-seq atlas, which assembles results from more than 400 independent published studies and encompasses more than 19 million immune cells from PBMCs and primary tumors, serves as an orthogonal cross-tissue annotation resource that supports the cell-type identity assignments made on the CyTOF data and extends cross-tissue comparisons. The scRNA-seq atlas is not used as a per-patient statistical comparator and does not contribute to any of the inferential analyses presented in this study. Immune cell types, including T cells, B cells, myeloid cells, and natural killer (NK) cells, were gated according to the expression of specific markers, including CD3, CD19, ITGAM (CD11b), and NCAM1 (CD56), in CyTOF analyses (Figure S2).

**Figure 2:**
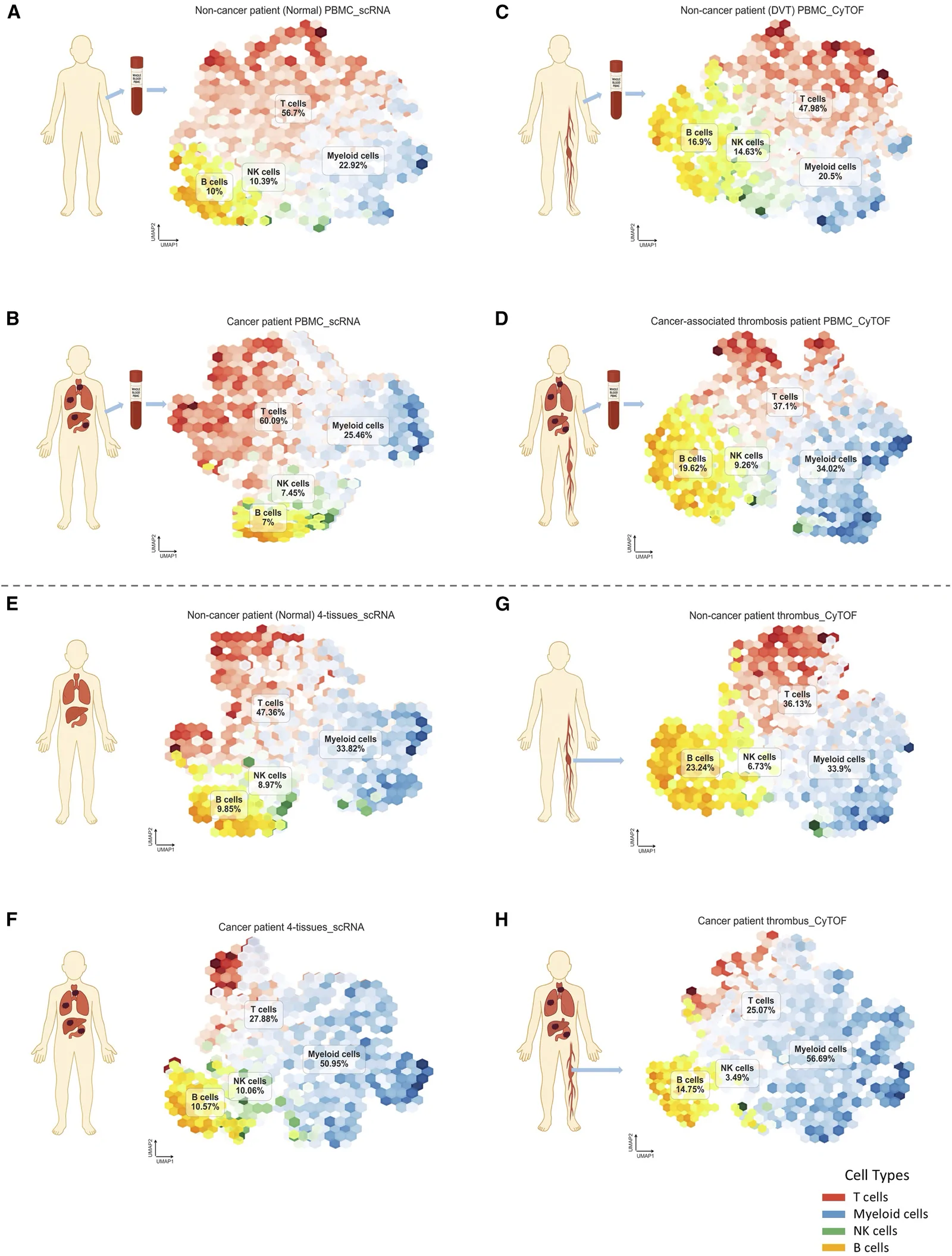
Global redistribution of major immune lineages across blood, thrombi, and cancer compartments UMAP projections depict single-cell landscapes colored by lineage identity (red, T cells; blue, myeloid cells; green, NK cells; and yellow, B cells) and annotated with lineage frequencies. PBMCs from (A) non-cancer and (B) cancer patients (liver, lung, stomach, and thyroid cancers) profiled by scRNA-seq. CyTOF profiles of PBMCs from (C) non-cancer patients and (D) cancer patients with DVT. scRNA-seq of (E) matched normal tissues and (F) primary tumor from the cancer cohort. CyTOF profiles of thrombus from (G) non-cancer and (H) cancer patients. In all figures, individual cells are colored according to their assigned major immune lineage (red for T cells, blue for myeloid cells, green for NK cells, and yellow for B cells). The color intensity within each lineage color does not encode an additional quantity but reflects the cell-by-cell density of points in the rendered embedding, with overlapping cells producing visually darker regions. scRNA-seq, single-cell RNA sequencing; CyTOF, mass cytometry; PBMCs, peripheral-blood mononuclear cells; DVT, deep-vein thrombosis.

In PBMC panels, scRNA-seq profiling revealed a modest increase in the proportion of myeloid cells in patients with cancer compared to normal individuals (25.46% vs. 22.92%), accompanied by a slight reduction in NK cells (Figures 2A and 2B). Notably, T cells remained the dominant PBMC lineage in both cancer and non-cancer subjects without thrombosis, accounting for 60.09% and 56.7% respectively (Figures 2A and-2B). Furthermore, CyTOF analysis demonstrated a significant alteration in immune cell composition in PBMCs from cancer patients with thrombosis compared to non-cancer patients with thrombosis, characterized by a marked decrease in T cells (37.1% vs. 47.98%) and NK cells (9.26% vs. 14.63%), along with a pronounced expansion of myeloid cells (34.02% vs. 20.50%; Figures 2C and 2D, and S3F). CyTOF analysis of thrombus samples from patients with cancer demonstrated a pronounced enrichment of myeloid cells compared to non-CAT (56.69% vs. 33.9%), coupled with a substantial reduction in T cell frequency (25.07% vs. 36.13%; Figures 2G and 2H). Consistent results were observed in scRNA-seq profiling of tissue samples, which similarly revealed a marked elevation of myeloid cell proportions in cancer tissues relative to normal tissues (50.95% vs. 33.82%) alongside a significant reduction in T cells (27.88% vs. 47.36%; Figures 2E and 2F). Although CyTOF analyses indicated notable reductions in both NK cells (3.49% vs. 6.73%) and B cells (14.75% vs. 23.24%) in CAT (Figures 2G and 2H), these trends were less apparent in scRNA-seq data from cancer tissues (NK cells: 10.06% vs. 8.97%; B cells: 10.57% vs. 9.85%; Figures 2E and 2F), suggesting potential modality-specific detection sensitivities or biological heterogeneity across compartments. Collectively, our multi-omics data support a model in which CAT involves dual-layer immune reprogramming, comprising systemic immunosuppression and a myeloid-rich, T cell-depleted microenvironment within thrombi.

### T cell subset remodeling in CAT

To further characterize the cellular features associated with CAT, we analyzed the distribution of T cell subsets in peripheral blood and thrombus samples from patients with or without cancer. We extracted 1 × 10^4^ CD45^+^ cells from each sample, and concatenated samples were displayed as t-distributed stochastic neighbor embedding (t-SNE) maps (Figure S3A). Marker t-SNE was employed to map the expression of all samples (Figure S3B). The differences in immune compartments were identified and calculated in each group (Figures S3C and S3D). Initial comparisons between blood and thrombus samples revealed a significant reduction in CD8^+^ and CD4^+^ T cells within thrombi (Figure S3D). In contrast, CD4^−^CD8^−^ double-negative (DN) T cells were significantly enriched at thrombotic samples (Figure S3D). Further stratification by cancer status revealed that these alterations were significantly accentuated in patients with cancer (Figures S3E and S3F). CD8^+^ T cells declined progressively from PBMCs to thrombi, accompanied by a pronounced reduction of CD4^+^ T cells within CAT (Figure S3F). In contrast, CD4^−^CD8^−^ DN T cells were dramatically expanded in CAT, emerging as the dominant subset at the local immune niche. These observations support a model in which CAT is accompanied by a loss of canonical effector of CD8^+^ and CD4^+^ helper T cells from thrombi and a reciprocal enrichment of unconventional CD4^−^CD8^−^ DN T cell populations.

### Immune-cell signatures in blood and thrombi differentiate CAT from non-cancer DVT

**Figure 3:**
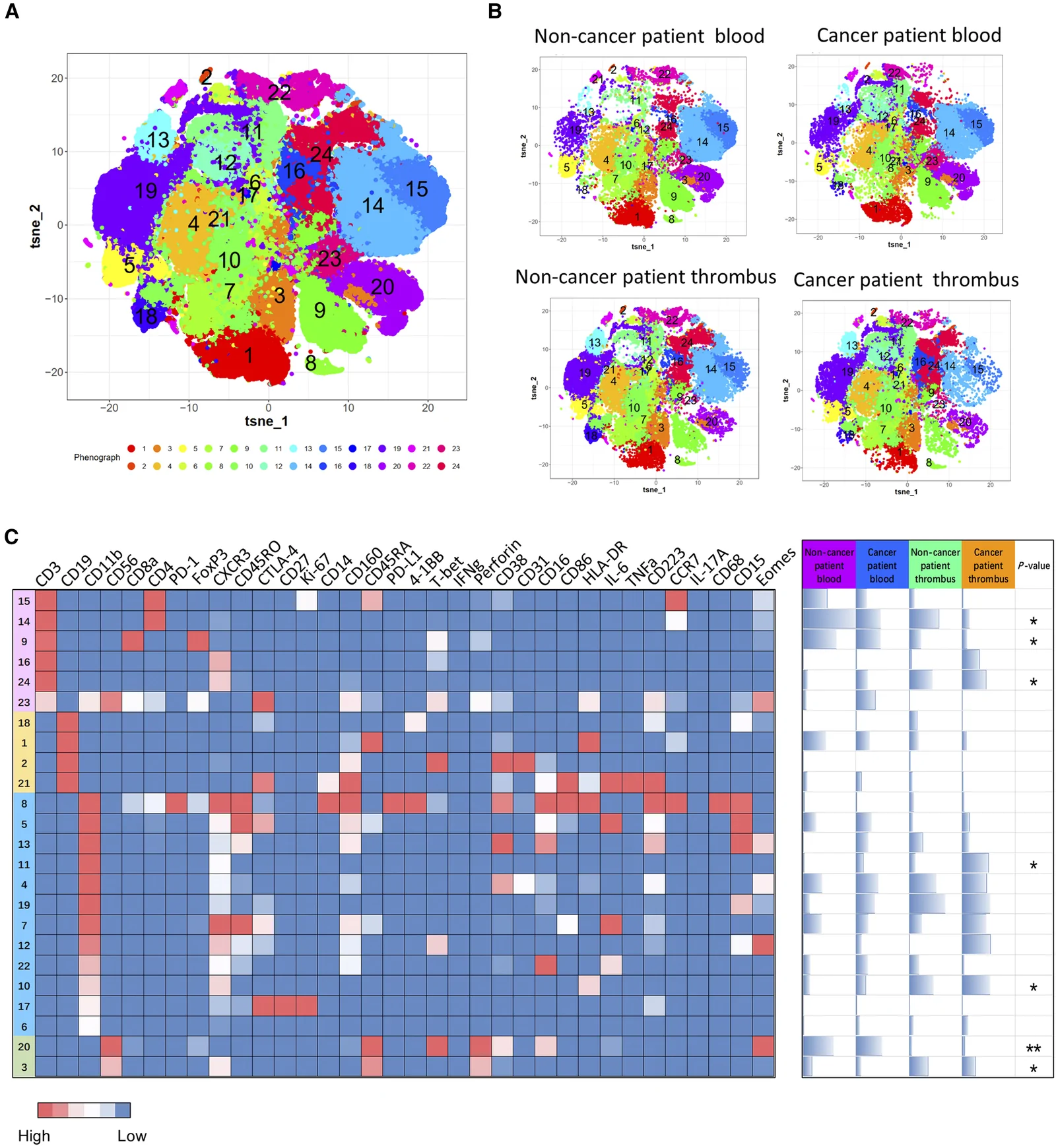
Phenotypic deconvolution of circulating and thrombus-resident immune populations (A) t-distributed stochastic neighbor embedding (t-SNE) of live CD45^+^ events pooled from all patients. Unsupervised phenograph clustering delineated 24 immune metaclusters, color-coded as indicated. (B) Group-wise t-SNE overlays reveal the redistribution of metaclusters in non-cancer patient blood, cancer patient blood, non-cancer DVT thrombus, and CAT thrombus. (C) Heatmap of scaled median marker intensity (row-wise *Z* score: blue = low and red = high) for 34 immune genes, activation, and exhaustion markers across the 24 metaclusters. Kernel-density bars show metacluster frequencies in each compartment. Statistical significance of frequency differences among the four compartments was assessed by one-way ANOVA followed by Benjamini-Hochberg-corrected Tukey’s post hoc test; ∗*p* < 0.05, ∗∗*p* < 0.01.

Dimensionality reduction using t-SNE revealed 24 phenotypically distinct immune cell clusters spanning both lymphoid and myeloid compartments (Figures 3A–3C). Memory and effector T cell compositions differed significantly between non-cancer DVT thrombi and CAT thrombi (Figure S4). Furthermore, CD8^+^ T cells, uniquely characterized by co-expression of FOXP3 and PRF1 (Perforin), showed significant enrichment in blood samples from patients with cancer relative to those with CAT (Figure S4), with this enrichment being particularly pronounced in samples from patients with lung cancer compared to other cancer types (Figure S5). The EOMES^+^TBX21^+^ (T-bet^+^) NK cell subset showed significant enrichment in blood samples from both cancer and non-cancer patients compared to thrombus samples (CAT and non-CAT; Figures 3C, S4B, and S4C). Functional marker analysis revealed elevated expression of exhaustion markers LAG3 and EOMES in T cells from blood of a patient with cancer compared to non-cancer blood (Figure S5), suggesting immune exhaustion of T lymphocytes in patients with cancer.

We identified four CD19^+^ B cell subtypes with distinct surface marker patterns (Figure 3C). Notably, the cells showed significantly higher CXCR3 expression in both non-CAT and CAT compared to blood samples (Figures S5C–S5E), suggesting an enrichment of activated B cells in thrombi. Compared with non-CAT, two myeloid subsets were notably more abundant in CAT, especially in lung cancer thrombi (Figures S4B and S5B). One subset expressed CD11b and CXCR3, indicating strong migratory and transport potential, along with HLA-DR expression. The other, characterized by PD-1 and PD-L1 expression, likely represents populations with a tumor-associated macrophage (TAM)- or myeloid-derived suppressor cell (MDSC)-like surface phenotype. Peripheral blood from patients with cancer was enriched for exhausted-like T cell phenotypes (PD-1^+^, EOMES^+^, and LAG3^+^) and depleted of central-memory CD4^+^ T cells compared with blood from non-cancer individuals (Figures S4A and S5F), indicating early systemic immune reprogramming. Non-CAT maintained a balanced mix of lymphoid and myeloid cells, whereas CAT contained approximately twice as many CXCR3-high myeloid cells (Figures 3C and S5C). The relative enrichment of these myeloid populations in CAT thrombi, together with their carriage of surface markers canonically associated with suppressive capacity, is consistent with a phenotypically immunosuppressive thrombus microenvironment, while direct functional confirmation through suppression assays will be required before this can be characterized as functionally immunosuppressive.

### Distinct immune remodeling from circulation to thrombi in CAT versus non-cancer DVT

**Figure 4:**
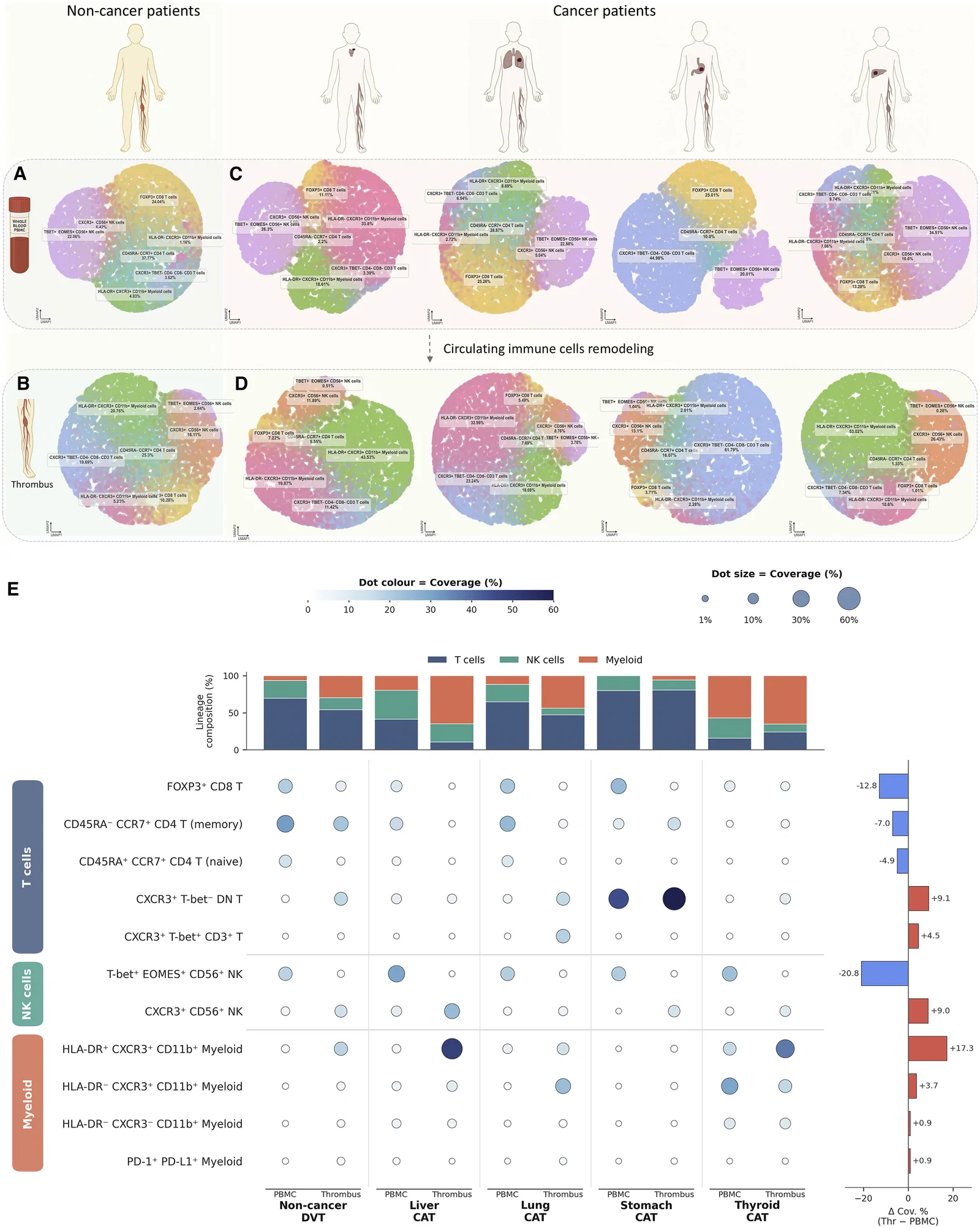
Immune subset remodeling in blood and thrombi across non-cancer DVT and CAT (A–D) High-dimensional CyTOF UMAP plots of paired PBMC and thrombus samples from non-cancer DVT (A and B) and CAT (C and D), stratified by cancer type (liver, lung, stomach, and thyroid). PBMC from non-cancer DVT (A) and CAT (C) serve as systemic baselines, while their matched thrombi (B and D) show pronounced myeloid expansion and depletion of NK, regulatory, and memory T cells, with cancer-type-associated patterns in CAT. (E) Bubble heatmap integrating coverage % of 11 phenograph-defined CD45^+^ clusters across all 10 disease-compartment groups (5 disease groups × PBMC/thrombus). Dot size and color encode coverage %, and clusters are grouped by lineage (T, NK, and myeloid). The top marginal shows a stacked bar of lineage composition (T cells, NK cells, and myeloid) per group. The right marginal shows, for each cluster, the thrombus-PBMC coverage difference averaged across the five disease groups, with red indicating thrombus-enriched and blue PBMC-enriched clusters. Non-cancer DVT, *n* = 5; lung CAT, *n* = 4; the liver, the stomach, and the thyroid CAT, *n* = 1 each. Cancer-type-stratified panels are descriptive only given limited per-cancer-type sample sizes.

To characterize immune landscape alterations associated with thrombus formation, we conducted CyTOF-based immunophenotyping by matching PBMC samples with thrombus samples from non-CAT patients or CAT patients across multiple cancer types. Significant immune remodeling was observed from PBMCs to thrombus samples, reflecting distinct adaptations within thrombotic immune microenvironments. In non-CAT, immune remodeling was characterized by a prominently enhanced infiltration or localized expansion of CXCR3^+^CD56^+^ NK cells (6.42% in PBMC vs. 16.11% in thrombi), HLA-DRA^+^CXCR3^+^ myeloid cells (4.93% in PBMC vs. 20.76% in thrombi), and CXCR3^+^T-bet^-^CD4^−^CD8^−^ DN T cells (3.62% in PBMC vs. 19.69% in thrombi; Figures 4A and 4B). Conversely, significant reductions in regulatory FOXP3^+^ CD8 T cells (24.04%–10.28%) and memory CD45RA^−^CCR7^+^ CD4 T cells (37.77%–25.30%) underscored a shift toward a more activated innate immune profile in thrombi. These findings suggest that the formation of non-CAT distinctly reshapes the local immune microenvironment by enhancing certain NK and myeloid subsets while significantly reducing regulatory T cell and specific memory T cell populations.

We then examined sub-immune cell profiles in PBMC across the four CAT cancer types represented in our cohort, recognizing that single-patient observations (liver, gastric, and thyroid) cannot be generalized and are presented here as descriptive signals only. In the single liver CAT case, PBMC contained the highest proportion of CXCR3^+^CD56^+^ NK cells (10.6%). Across the four lung CAT samples, PBMC showed elevated CD45RA^−^CCR7^+^ CD4 T cells (mean: 28.87%). The single gastric CAT case showed a notable abundance of FOXP3^+^ CD8 T cells (25.01%) and CXCR3^+^T-bet^-^CD4^−^CD8^−^ DN T cells (44.98%), and the single thyroid CAT case showed enrichment of HLA-DRA^-^CXCR3^+^ myeloid leukocytes (33.8%). These observations are consistent with the cancer-type heterogeneity of CAT documented in the broader literature and provide hypothesis-generating signals for future targeted studies, but should not be interpreted as cancer-type-specific statistical findings (Figures 4C and 4D).

**Figure 5:**
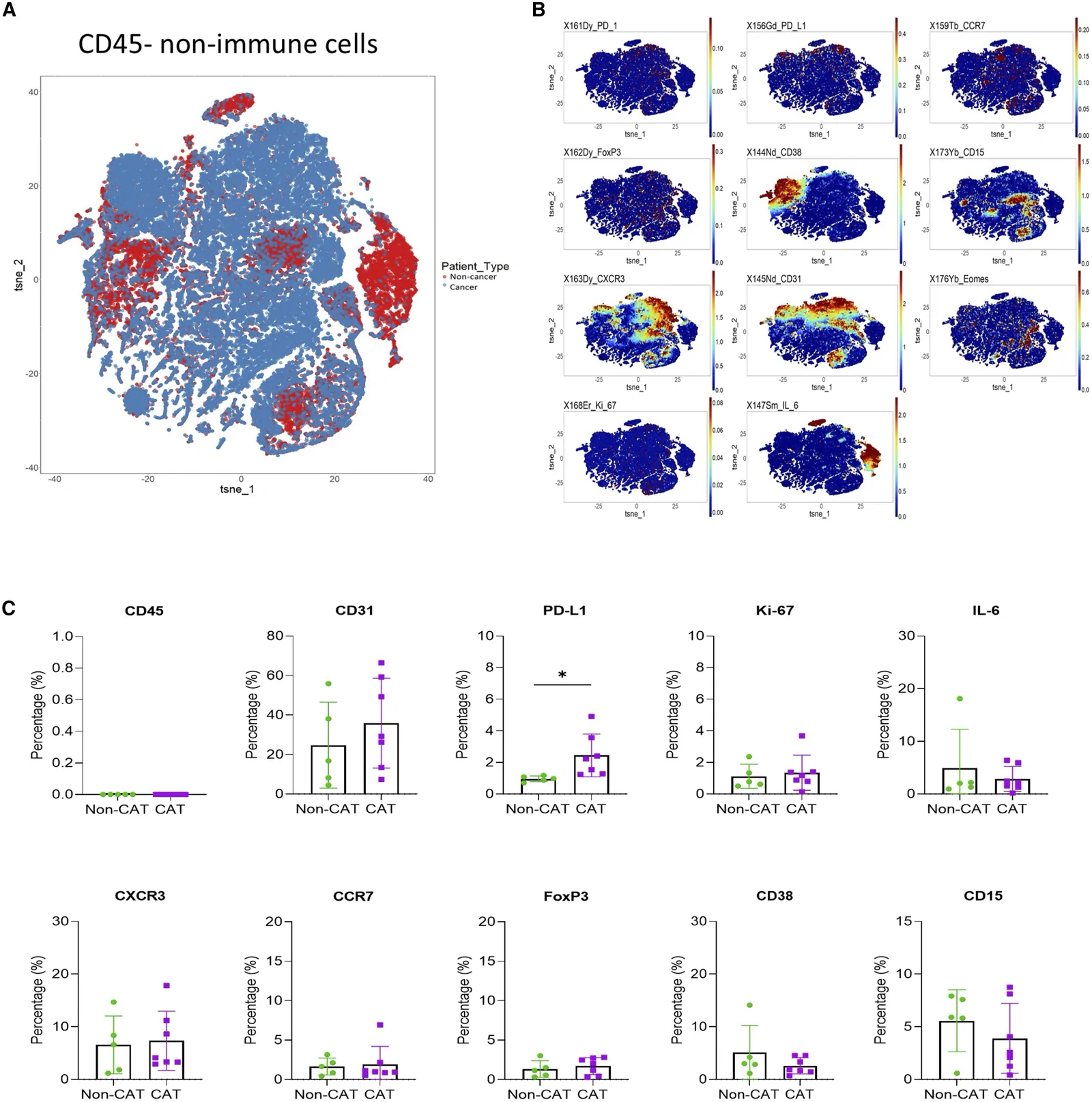
Selective enrichment of PD-L1 on the CD45^−^non-immune compartment in CAT thrombi (A) Two-dimensional t-SNE plot of CD45^−^ non-immune cells isolated from thrombus samples of patients with CAT (blue) and non-cancer DVT (red; *n* = 12 total patients). Each dot represents one individual cell. (B) Expression intensity of key non-immune and functional markers, including endothelial (CD31), immune-checkpoint (PD1 and PD-L1), chemokine receptors (CXCR3 and CCR7), proliferation (Ki-67), inflammation (IL-6), activation (CD38), granulocyte-related (CD15), and transcription factors (FoxP3 and Eomes), overlaid onto the t-SNE map. (C) Quantification of non-immune marker expression between non-cancer DVT and CAT thrombi. For panels stratified by cancer type, comparisons across thyroid, liver, gastric, and lung CAT are presented as descriptive comparisons of cluster frequencies given the limited per-cancer-type sample sizes, and any statistical annotations shown should be interpreted in this descriptive context. Bars represent mean ± SD; each dot indicates an individual patient. Statistical significance determined by two-tailed unpaired Student’s *t* test (∗*p* < 0.05).

In the matched CAT thrombus samples, related yet distinguishable patterns were observed across the four cancer types, with the caveat that thyroid, liver, and gastric CAT are each represented by a single patient and that all per-cancer-type changes are reported here as descriptive observations rather than inferential comparisons. In the single liver CAT case, the thrombus showed relative increases in CXCR3^+^CD56^+^ NK cells and HLA-DRA^+^CXCR3^+^ myeloid cells compared with the matched PBMCs, alongside relative reductions in T-bet^+^EOMES^+^CD56^+^ NK cells and FOXP3^+^ CD8 T cells. Across the four lung CAT samples, thrombi showed relative increases in HLA-DRA-CXCR3^+^ myeloid leukocytes, PD-1^+^PD-L1^+^ myeloid cells, and CXCR3^+^T-bet^+^ T cells, together with relative reductions in FOXP3^+^ CD8 T cells and T-bet^+^EOMES^+^CD56^+^ NK cells. In the single gastric CAT case, the thrombus showed relative increases in CXCR3^+^T-bet^-^CD4^−^CD8^−^ DN T cells and CXCR3^+^CD56^+^ NK cells alongside a relative reduction in FOXP3^+^ CD8 T cells, while in the single thyroid CAT case the thrombus showed relative increases in HLA-DRA^+^CXCR3^+^ myeloid cells and CXCR3^+^T-bet^-^CD4^−^CD8^−^ DN T cells with a relative reduction in T-bet^+^EOMES^+^CD56^+^ NK cells (Figures 4C and 4D). These patterns are summarized across all ten disease-compartment groups (Figure 4E), which also displays lineage composition per group (top marginal) and the thrombus-PBMC coverage difference for each cluster (right marginal). The scRNA-seq data from these four types of primary cancer demonstrated similar cell subset patterns to those in gastric cancer thrombi (Figure S6). To assess non-immune contributions to the thrombus immune microenvironment, we extracted CD45^−^ non-immune cells from each specimen in CyTOF data and profiled them (Figures 5A and 5B). The paired analysis revealed a significant increase in PD-L1-expressing CD45^−^ non-immune cells in CAT (Figure 5C). This selective PD-L1 up-regulation suggests that, beyond hematopoietic remodeling, the CD45^−^ non-immune compartment in CAT thrombi shows checkpoint enrichment that may contribute to local immune modulation, although the present panel does not allow unambiguous assignment of these PD-L1^+^ events to a specific cellular origin (endothelial, true stromal, or, in CAT, potentially epithelial or tumor derived).

### Exploratory correlation analysis between hematological biomarkers and immune-cell subsets in PBMC and thrombus samples

We first compared routine hematological and biochemical profiles between CAT and non-cancer DVT patients in the broader hematology cohort (Table S2) to identify potential clinical confounders. The subsequent exploratory regression analyses linking these clinical indices to immune-cell proportions were performed on the subset of 10 patients for whom both CyTOF profiling and biochemistry were available. The exploratory framing, the per-tissue and combined paired-sample analyses, and the Benjamini-Hochberg false discovery rate (BH-FDR) correction are detailed in the STAR Methods and in Tables S4, S5A, and S5B. The significant differences were observed in hemoglobin, carcinoembryonic antigen (CEA), and chloride levels. However, there is no significant difference for other parameters evaluated in routine hematological and biochemical tests between the CAT and non-CAT patient groups (Table S2). However, significant differences were observed in hemoglobin (cancer group median: 99.0 g/L vs. non-cancer group median: 118.0 g/L, *p* = 0.042), CEA levels (cancer group median: 3.7 μg/L vs. non-cancer group median: 2.0 μg/L, *p* = 0.049), and chloride (cancer group median: 99.3 mmol/L vs. non-cancer group median: 103.7 mmol/L, *p* = 0.042; Table S2). These findings indicate that lower hemoglobin, chloride, and elevated CEA levels may serve as distinguishing characteristics for CAT patients. We then fitted exploratory univariable regression models adjusted for cancer status. After BH-FDR correction within each immune-cell subset, a positive association between hemoglobin and FOXP3^+^CD8^+^ T cell frequency was identified as the most robust signal in our exploratory analysis. This association survived FDR correction in both compartments examined separately (PBMC: β = 0.673, 95% confidence interval [CI] 0.400–0.946, raw *p* < 0.001, BH-FDR *q* = 0.005, Table S5A; thrombus: β = 0.427, 95% CI 0.172–0.681, raw *p* = 0.005, BH-FDR *q* = 0.043, Table S4). Crucially, in the combined paired-sample model that integrated PBMC and thrombus observations from the same patients (Table S5B), this association was further strengthened (β = 0.550, raw *p* < 0.0001, BH-FDR q < 0.001), and a directionally consistent positive association between FOXP3^+^CD8^+^ T cells and RBC counts also survived FDR correction (β = 0.441, raw *p* = 0.001, BH-FDR *q* = 0.006). Additional associations reaching nominal significance (raw *p* < 0.05) but not surviving FDR correction included an inverse correlation between thrombocyte count and HLA-DRA^-^CXCR3^+^ myeloid leukocytes in thrombus and inverse correlations between WBC or neutrophil counts and T-bet^+^EOMES^+^CD56^+^ NK cells in thrombus (Table S4); these are reported as nominal, hypothesis-generating signals only. Collectively, the exploratory analysis identifies erythroid-lineage indices, particularly hemoglobin, as positively associated with FOXP3^+^CD8^+^ regulatory T cell frequencies across both PBMC and thrombus compartments, while the platelet- and granulocyte-related thrombus-restricted signals require validation in larger, adequately powered cohorts.

## Discussion

Our study provides an integrated single-cell immune profiling of CAT, revealing distinct immune remodeling between the systemic circulation and the local thrombus microenvironment. By combining high-dimensional CyTOF and scRNA-seq analyses across matched peripheral blood, thrombus samples, primary tumors, and normal tissues, we identified distinct yet interconnected immunological changes that are associated with thrombus formation and progression in patients with cancer, offering correlative insights with translational potential.

We initially observed pronounced systemic immune dysregulation characterized by marked myeloid cell expansion and concurrent T and NK cell depletion in peripheral blood from patients with cancer, a phenotype strongly associated with heightened expression of markers associated with an exhausted-like phenotype (PD-1^+^, EOMES^+^, and LAG3^+^). This finding aligns with previous studies reporting impaired immune surveillance and T cell exhaustion in the cancer context, and identifies systemic immune dysfunction as a feature associated with CAT.^19^^,^^20^^,^^21^ Focusing on thrombus samples, we observed a striking enrichment of myeloid cells, particularly CXCR3^+^CD11b^+^ subsets, in CAT thrombi compared with non-cancer DVT thrombi. These myeloid subsets carry surface and transcriptional features previously associated with immunosuppression and chronic inflammation, and the present findings are consistent with an immunosuppressive and inflammatory marker profile in the thrombus microenvironment. The signal was most pronounced in lung CAT, consistent with prior literature describing myeloid populations associated with immune suppression in this cancer type.^22^

Within thrombotic microenvironment, the T cell compartment underwent notable remodeling, characterized by substantial depletion of classical CD4^+^ helper and CD8^+^ cytotoxic T cells alongside a pronounced enrichment of unconventional CD4^−^CD8^−^ DN T cells. Although DN T cells have been previously implicated in immune regulation and chronic inflammatory conditions, their specific roles and functional implications in CAT warrant future investigation and may suggest candidate populations for hypothesis-driven immunological studies.^23^^,^^24^ Interestingly, we identified a unique population of CD8^+^ T cells co-expressing FOXP3 and perforin (FOXP3^+^ PRF1^+^), particularly enriched in peripheral blood from lung CAT patients. The dual regulatory and cytotoxic signature suggests complex functions, potentially reflecting a compensatory response to cancer-associated systemic immune dysregulation. Our findings are consistent with a nuanced coupling between systemic circulation and locally observed immune modulation in CAT, and dedicated functional assays will be required to define the suppressive and cytotoxic capacities of this subset.^25^

Our comparative analysis across cancer types revealed distinct systemic and intrathrombus immune signatures, consistent with cancer-specific immune imprinting. For example, in lung CAT, we observed a significant expansion of populations with an MDSC-like surface phenotype, with high PD-L1 expression and, in a subset, detectable PD-1. These populations are associated with attenuation of effector T cell responses and with tumor immune evasion.^26^ Simultaneously, we observed consistent depletion of FOXP3^+^ CD8^+^ regulatory T cells and memory CD4^+^ T cells across multiple CAT, further highlighting factors associated with cancer in this cohort. Furthermore, our analysis identifies elevated PD-L1 expression on the CD45^−^ non-immune compartment in CAT thrombi, a previously under-recognized observation. The CD45^−^ compartment of thrombus tissue is heterogeneous and includes endothelial cells (CD31^+^), true stromal cells, and, in CAT, potentially epithelial or tumor-derived cells, and the present panel cannot unambiguously assign the PD-L1 signal to a specific cellular origin. Nonetheless, the observation suggests an additional checkpoint-rich layer outside the hematopoietic compartment that may be associated with local immune modulation in CAT thrombi.^27^ Additionally, NK cell subsets marked by T-bet^+^ and EOMES^+^ expression were expanded in blood from non-cancer DVT and are associated with maintenance of mature cytotoxic NK programs.^28^ Our findings align with preclinical data showing that CXCR3 expression is essential for NK cell recruitment into cancer sites, as CXCL10 gradients guide the migration.^29^ The observed increases in CXCR3^+^ NK cells in selected CAT contexts may reflect an activated, yet insufficient, innate response to malignant cells or to endothelial activation within thrombi.

Clinically, the most robust association in our exploratory analysis was a positive correlation between hemoglobin and FOXP3^+^CD8^+^ T cells in PBMC, with a directionally consistent but nominal trend in thrombus. Additional nominal associations between platelet/neutrophil counts and intrathrombus NK and myeloid populations did not survive multiple testing correction and are reported as exploratory, hypothesis-generating signals only. FOXP3^+^ CD8^+^ regulatory T cells represent a bona fide immunoregulatory lineage with established roles in attenuating neutrophil-driven inflammation and stabilizing hematopoiesis.^30^ Our findings highlight previously unrecognized hematological-immune correlations that may be associated with thrombus formation and progression, although the small cohort size, the cross-sectional design, and the exploratory nature of these analyses preclude conclusions about directionality between systemic factors and the local thrombus environment, and validation in adequately powered cohorts will be required.

**Figure 6:**
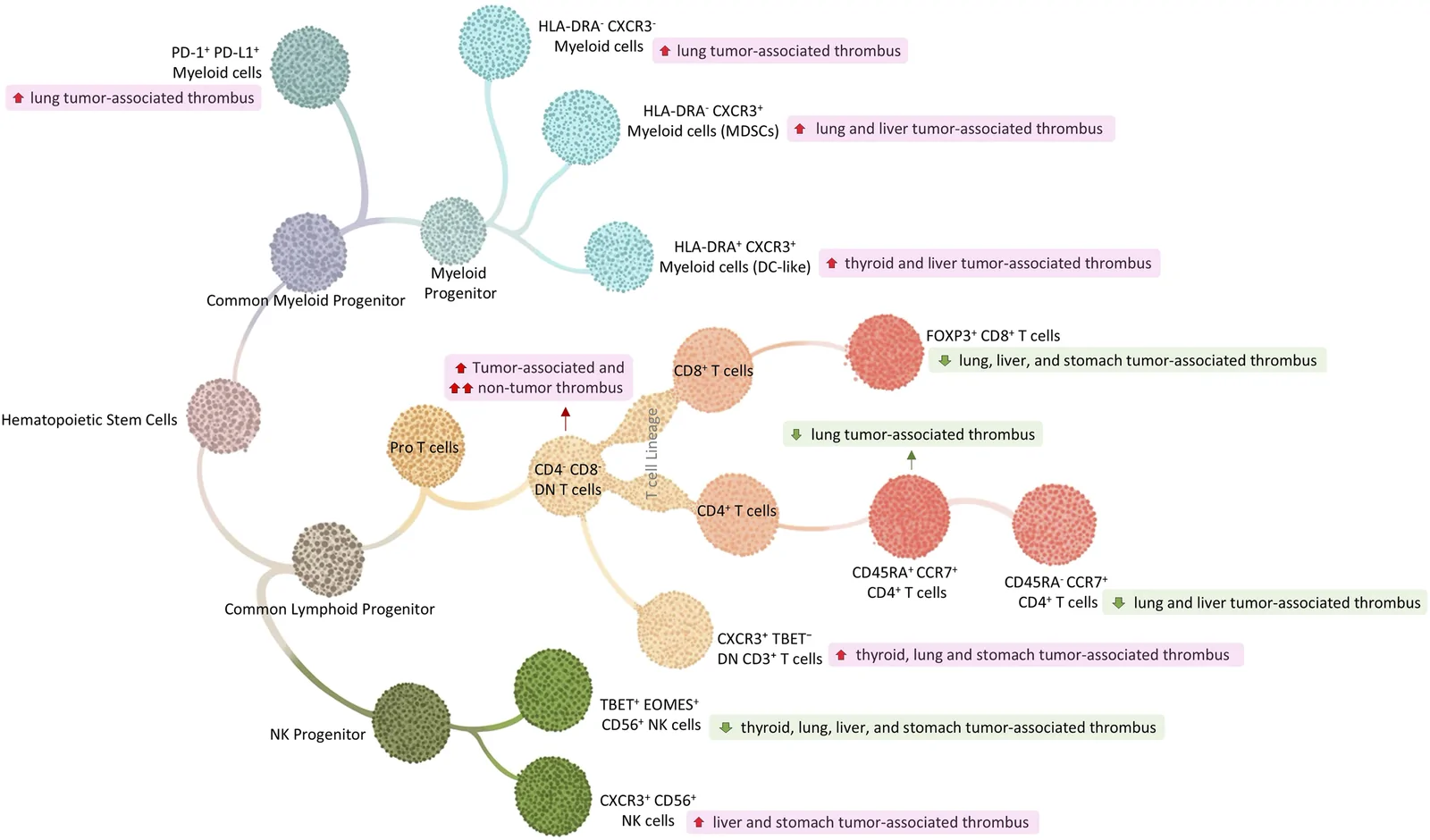
Lineage-trajectory map summarizing cancer-type-associated immune remodeling in thrombus niches Immune cells isolated from thrombi and paired blood were embedded in a force-directed developmental graph constructed from canonical hematopoietic trajectories. Arrows and colored labels annotate major changes in abundance for specific cell subsets in CAT compared to non-cancer DVT, and further specify the cancer types in which these changes were observed in lung, liver, stomach, and thyroid cancers. Upward magenta arrows indicate enrichment in CAT; downward green arrows indicate depletion. The figure represents a working model and a hypothesis-generating framework rather than a validated mechanistic map; directional and abundance annotations summarize correlative observations from high-dimensional phenotypic profiling, and the proposed pathway-level interactions and directional assignments require future experimental validation through functional assays and *in vivo* modeling.

In conclusion, our integrative single-cell analysis delineates distinct and complex immune remodeling associated with CAT, characterized by prominent myeloid expansion accompanied by an immunosuppressive marker profile and by lymphoid subset reorganization. Specifically, we observed a marked relative enrichment of myeloid subsets carrying surface markers canonically associated with suppressive capacity, notably PD-1^+^PD-L1^+^ and HLA-DRA^-^CXCR3^+^ populations, particularly in lung and thyroid CAT. Concurrently, the lymphoid compartment showed a relative enrichment of unconventional CXCR3^+^T-bet^-^CD4^−^CD8^−^ DN T cells and a relative reduction in T-bet^+^EOMES^+^ NK cells and FOXP3^+^CD8^+^ T cells with a regulatory-like phenotype across multiple CAT, consistent with cancer-type-associated patterns of immune remodeling (Figure 6). Collectively, our study highlights dual-level, systemic and local immune dysregulation in CAT and provides a correlative atlas with translational implications, including testable hypotheses around local PD-1/PD-L1 checkpoint modulation within thrombi, myeloid-directed reprogramming, and restoration of CXCR3-dependent NK trafficking, all of which require dedicated functional validation in adequately powered studies.

### Limitations of the study

Several limitations should be acknowledged. The cohort is small, reflecting the rarity of paired thrombus and PBMC material from surgical thrombectomy and the resource intensity of dual-platform CyTOF and scRNA-seq profiling. Although the DVT-versus-CAT primary comparison is adequately powered to detect aggregate differences, all findings should be regarded as exploratory and require confirmatory validation in larger, multi-center cohorts. Cancer-type-specific observations derive from single patients for thyroid, liver, and stomach CAT and are descriptive only. In addition, although the CyTOF panel includes CD31 and therefore identifies endothelial cells within the CD45^−^ compartment, it lacks canonical epithelial, fibroblastic, and tumor-cell markers, so the cellular origin of the PD-L1 signal in the CD45^−^ non-immune compartment cannot be resolved. Finally, marker-based phenotypic annotations are inferred from canonical surface and transcription-factor markers and have not been validated by orthogonal functional assays such as cytotoxicity or suppression assays; these annotations should therefore be interpreted as phenotypic rather than functional designations. Future studies incorporating *in vitro* functional assays and *in vivo* models will be required to test the hypotheses generated here.

## Resource availability

### Lead contact

Requests for further information and resources should be directed to and will be fulfilled by the lead contact, Chen-Wei Yu (155342@mail.fju.edu.tw).

### Materials availability

No new unique reagents were generated in this study. All antibodies and reagents used in the CyTOF panel are commercially available, as listed in the key resources table.

### Data and code availability

The raw mass cytometry (.fcs) data, panel information, and per-patient sample metadata for the 12 CyTOF patients reported in this study, together with a cluster-composition summary table reporting cell-type coverage percentages for the CyTOF and scRNA-seq analyses, have been deposited at Mendeley Data (DOI: https://doi.org/10.17632/phybydwmb8.3) and are publicly available as of the date of publication. Reviewer access during the peer-review period is provided through the embargoed-link mechanism of the Mendeley Data platform. The scRNA-seq data underlying the cross-tissue comparisons were retrieved from the BioTuring Talk2Data v5 platform (https://bioturing.com/); this is an external resource governed by the BioTuring data-access terms, and the corresponding raw scRNA-seq matrices are not redeposited here. All original analysis code, including the R-based CyTOF preprocessing pipeline, the Phenograph clustering parameters, and the Python regression and figure-generation scripts, have been deposited at the same Mendeley Data record (https://doi.org/10.17632/phybydwmb8.3) and are publicly available as of the date of publication. Any additional information required to reanalyze the data reported in this paper is available from the lead contact upon request.

## Acknowledgments

This study was carried out in collaboration with BioTuring Inc. (San Diego, CA, USA), which provided access to curated single-cell RNA-sequencing datasets and bioinformatic support. We are deeply indebted to Prof. Qiuyu Zhang (Institute of Immunotherapy, Fujian Medical University) and Prof. Lieping Chen (Department of Medicine, Yale University) for their generosity in providing the necessary instrumentation and a supportive research environment. This study was supported by grants from the Joint Funds for the Innovation of Science and Technology, Fujian province, China (grant no. 2024Y9498), and the MOST 112-2314-B-030-002-MY3, Taiwan.

## Author contributions

L.Z., writing – original draft, data curation, and visualization; Y.X., writing – original draft, data curation, and supervision; B.C., methodology; X.L., methodology; H.-C.Y., methodology and supervision; C.-W.Y., writing-review and editing, supervision, funding acquisition, and conceptualization.

## Declaration of interests

The authors declare no competing interests.

## STAR★Methods

### Key resources table

**Table:** 

REAGENT or RESOURCE	SOURCE	IDENTIFIER
**Antibodies**		

Anti-Human CD45 (89Y), Clone HI30	Standard BioTools (formerly Fluidigm)	Cat# 3089003B; RRID: AB_2661851
Anti-Human CD19 (142Nd), Clone HIB19	Standard BioTools (formerly Fluidigm)	Cat# 3142001B; RRID: AB_2651155
Anti-Human CD38 (144Nd), Clone HIT2	Standard BioTools (formerly Fluidigm)	Cat# 3118012B; RRID: AB_314354
Anti-Human CD31 (145Nd), Clone WM59	Standard BioTools (formerly Fluidigm)	Cat# 3145004B; RRID: AB_2737262
Anti-Human CD8a (146Nd), Clone RPA-T8	Standard BioTools (formerly Fluidigm)	Cat# 3146001B; RRID: AB_3661846
Anti-Human IL-6 (147Sm), Clone MQ2-13A5	Standard BioTools (formerly Fluidigm)	Cat# 3147002B; BRID: AB_2810623
Anti-Human CD16 (148Nd), Clone 3G8	Standard BioTools (formerly Fluidigm)	Cat# 3148004B; RRID: AB_2661791
Anti-Human CD56 (NCAM) (149Sm), Clone NCAM16.2	Standard BioTools (formerly Fluidigm)	Cat# 3149021B; RRID: AB_397180
Anti-Human CD86 (150Nd), Clone IT2.2	Standard BioTools (formerly Fluidigm)	Cat# 3150020B; RRID: AB_2687852
Anti-Human CD14 (151Eu), Clone M5E2	Standard BioTools (formerly Fluidigm)	Cat# 3151009B; RRID: AB_314184
Anti-Human TNF-α (152Sm), Clone Mab11	Standard BioTools (formerly Fluidigm)	Cat# 3152002B; RRID: AB_315253
Anti-Human CD45RA (155Gd), Clone HI100	Standard BioTools (formerly Fluidigm)	Cat# 3155011B; BRID: AB_2810246
Anti-Human CD274 (PD-L1) (156Gd), Clone 29E.2A3	Standard BioTools (formerly Fluidigm)	Cat# 3156026B; RRID: AB_2565429
Anti-Human CD137 (4-1BB) (158Gd), Clone 4B4-1	Standard BioTools (formerly Fluidigm)	Cat# 3158013B; RRID: AB_314781
Anti-Human CD197 (CCR7) (159 Tb), Clone G043H7	Standard BioTools (formerly Fluidigm)	Cat# 3159003A; RRID: AB_2563726
Anti-Human T-bet (160Gd), Clone 4B10	Standard BioTools (formerly Fluidigm)	Cat# 3160010B; RRID: AB_2563788
Anti-Human FOXP3 (162Dy), Clone PCH101	Standard BioTools (formerly Fluidigm)	Cat# 3162011A; RRID: AB_467554
Anti-Human CD183 (CXCR3) (163Dy), Clone G025H7	Standard BioTools (formerly Fluidigm)	Cat# 3163004B; RRID: AB_2563724
Anti-Human CD45RO (164Dy), Clone UCHL1	Standard BioTools (formerly Fluidigm)	Cat# 3164007B; RRID: AB_2563752
Anti-Human IFN-γ (165Ho), Clone B27	Standard BioTools (formerly Fluidigm)	Cat# 3165002B; RRID: AB_2562849
Anti-Human CD27 (167Er), Clone O323	Standard BioTools (formerly Fluidigm)	Cat# 3167002B; RRID: AB_2562817
Anti-Human Ki-67 (168Er), Clone B56	Standard BioTools (formerly Fluidigm)	Cat# 3168001B; RRID: AB_393778
Anti-Human IL-17A (169 Tm), Clone BL168	Standard BioTools (formerly Fluidigm)	Cat# 3169006B; RRID: AB_2566764
Anti-Human CD3 (170Er), Clone UCHT1	Standard BioTools (formerly Fluidigm)	Cat# 3170001B; RRID: AB_2661807
Anti-Human CD68 (171 Yb), Clone Y1/82A	Standard BioTools (formerly Fluidigm)	Cat# 3110017B; RRID: AB_1089058
Anti-Human CD4 (174 Yb), Clone SK3	Standard BioTools (formerly Fluidigm)	Cat# 3174004B; RRID: AB_2687862
Anti-Human Perforin (175Lu), Clone BD48	Standard BioTools (formerly Fluidigm)	Cat# 3175004B; RRID: AB_2571973
Anti-Human CD11b (Mac-1) (209Bi), Clone ICRF44	Standard BioTools (formerly Fluidigm)	Cat# 3209003B; RRID: AB_2687654
Anti-Human HLA-DR (141Pr), Clone L243	BioLegend (self-conjugated)	Cat# 307611; RRID: AB_314689
Anti-Human CD223 (LAG-3) (154Sm), Clone 7H2C65	BioLegend (self-conjugated)	Cat# 369202; RRID: AB_2616877
Anti-Human CD160 (153Eu), Clone BY55	BioLegend (self-conjugated)	Cat# 341202; RRID: AB_2074411
Anti-Human CD279 (PD-1) (161Dy), Clone M3	BioLegend (self-conjugated)	Cat# 329902; RRID: AB_940488
Anti-Human CD152 (CTLA-4) (166Er), Clone L3D10	BioLegend (self-conjugated)	Cat# 349931; RRID: AB_2783243
Anti-Human CD15 (173 Yb), Clone W6D3	BioLegend (self-conjugated)	Cat# 323002; RRID: AB_756008
Anti-Human Eomes (176 Yb), Clone 7C9B03	BioLegend (self-conjugated)	Cat# 662002; RRID: AB_2564182
Cisplatin viability reagent (Cell-ID Cisplatin-198Pt)	Standard BioTools	Cat# 201198
Cell-ID Intercalator-Ir (191Ir/193Ir)	Standard BioTools	Cat# 201192A
EQ Four Element Calibration Beads	Standard BioTools	Cat# 201078
Human TruStain FcX (Fc receptor blocking solution)	BioLegend	Cat# 422301
Foxp3/Transcription Factor Staining Buffer Set	BioLegend (or eBioscience equivalent)	Cat# 421403
Maxpar Antibody Labeling Kits (lanthanide isotopes)	Standard BioTools	Cat# 201141A, 201145A, 201148A, 201150A, 201151A, 201152A, 201154A, 201155A, 201159A, 201160A, 201161A, 201165A, 201166A, 201167A, 201168A, 201170A, 201172A, 201173A, 201175A, 201176A

**Biological samples**		

Human venous thrombi obtained at surgical thrombectomy from patients with non-cancer DVT (*n* = 5) and cancer-associated thrombosis (CAT; *n* = 7)	This paper (Fujian Provincial People’s Hospital, ethics 2025-006-01)	N/A
Matched human peripheral blood mononuclear cells (PBMC) from the same patients (*n* = 12 paired with thrombus)	This paper	N/A

**Critical commercial assays**		

Ficoll-Paque PLUS density gradient medium	Cytiva	Cat# 17144002
Collagenase IV	Worthington/Sigma-Aldrich	Cat# LS004189/C5138
DNase I	Sigma-Aldrich	Cat# 11284932001
GentleMACS Octo Dissociator	Miltenyi Biotec	Cat# 130-095-937

**Deposited data**		

Raw mass cytometry (.fcs) data, panel information, per-patient sample metadata, a cluster-composition summary table, and original analysis code for the 12 CyTOF patients reported in this study	This paper; Mendeley Data	https://doi.org/10.17632/phybydwmb8.3
Integrated single-cell RNA-sequencing atlas of immune cells from PBMC and primary tumors (>19 million cells from >400 published studies)	BioTuring Talk2Data v5 platform	https://bioturing.com/

**Software and algorithms**		

FlowJo (v10.0) and FlowJo CyTOF plugin	BD Life Sciences	https://www.flowjo.com; RRID:SCR_008520
R-based semi-automated CyTOF analysis pipeline (v 4.0.0); CATALYST-style workflow with Phenograph clustering and t-SNE	R Foundation; Phenograph: Levine et al., Cell, 2015	https://www.r-project.org/; CRAN
R (v 4.3.2) for statistical analyses (OLS regression, BH-FDR correction)	R Foundation	https://www.r-project.org/
Python (v 3.13.3) for visualization and downstream analysis of the integrated scRNA-seq atlas	Python Software Foundation	https://www.python.org/; RRID:SCR_008394
UMAP algorithm for the integrated scRNA-seq atlas embedding	McInnes, Healy, Melville (2018)	https://umap-learn.readthedocs.io/; RRID:SCR_018217
GraphPad Prism (v 10.0)	GraphPad Software	https://www.graphpad.com/; RRID:SCR_002798

**Other**		

Helios mass cytometer (CyTOF instrument)	Standard BioTools (formerly Fluidigm)	N/A

### Experimental model and study participant details

#### Human subjects

All experiments were reviewed and approved by the Institutional Review Board of Fujian Provincial People’s Hospital (Ethical approval number 2025-006-01). Written informed consent was obtained from all participants prior to enrollment. Adult human patients undergoing surgical thrombectomy for venous thrombosis at Fujian Provincial People’s Hospital were prospectively enrolled. The CyTOF cohort comprised 12 patients providing 24 paired specimens (12 venous thrombi and 12 matched PBMC samples), divided into a non-cancer deep vein thrombosis (DVT) group (*n* = 5 patients, 10 specimens) and a cancer-associated thrombosis (CAT) group (*n* = 7 patients, 14 specimens). Within the CAT group, primary cancers comprised lung (*n* = 4), thyroid (*n* = 1), liver (*n* = 1), and gastric (*n* = 1) malignancies. All CAT patients were treatment-naive at sampling (no prior systemic chemotherapy, radiotherapy, or immunotherapy). Demographic information (sex and age) was available for 10 of the 12 patients (3 non-cancer DVT and all 7 CAT); it was not recorded for two non-cancer DVT patients (Patients 2 and 11). Among these 10 patients, 9 were male and 1 was female, with a median age of 66.5 years (range 32–77). The cohort was predominantly male (9 of 10 patients with recorded sex), precluding assessment of sex effects on the reported phenotypes. The integrated scRNA-seq atlas, drawn from the BioTuring Talk2Data v5 platform encompassing more than 19 million immune cells from more than 400 independent published studies, was used as an orthogonal cross-tissue annotation resource and is not used as a per-patient statistical comparator. The clinical and biochemical comparison cohort presented in Table S2 represents a broader population of 25 patients (12 non-cancer DVT and 13 CAT) for whom routine laboratory parameters were available. Per-patient platform availability is detailed in Table S1, and an overview of cross-platform sample numbers is provided in Table S6. This study did not use any cell lines.

#### Cohort design and statistical considerations

This study was designed as a pilot atlas resource for CAT. Two practical realities of high-dimensional single-cell immunology in human thrombus material informed this design: thrombus tissue from patients undergoing surgical thrombectomy is rare and not amenable to routine collection, particularly when paired with matched PBMC and full clinical annotation, and dual-platform CyTOF and scRNA-seq cross-referencing is highly resource-intensive. Comparable landmark single-cell studies of human thrombi and tumor microenvironments typically profile 5 to 15 patients per arm. Because no prior effect-size estimates exist for thrombus-resident immune subsets in CAT, a formal *a priori* power calculation was not feasible. Accordingly, the primary statistical comparison contrasts non-cancer DVT (*n* = 5) with CAT (*n* = 7) at the aggregate level, where formal hypothesis testing remains scientifically appropriate to identify the shared CAT-associated immune signature. Cancer-type-specific findings are presented as descriptive, hypothesis-generating observations rather than inferential comparisons, given the limited per-cancer-type sample sizes (*n* = 1 for thyroid, liver, and gastric CAT). All findings, including those reaching nominal statistical significance in the DVT-vs-CAT comparison, are exploratory observations requiring confirmatory validation in adequately powered, multi-center cohorts.

### Method details

#### Thrombus dissociation and PBMC isolation

Venous thrombi were collected at the time of surgical thrombectomy in chilled RPMI-1640 medium (Gibco) and processed within 2 h. Thrombi were minced into ∼1-mm fragments using sterile scissors and digested in RPMI-1640 supplemented with DNase I (200 U/mL; Sigma-Aldrich) and Collagenase IV (1 mg/mL; Worthington/Sigma-Aldrich) using the GentleMACS Octo Dissociator (Miltenyi Biotec) at 37 °C for 1 h with intermittent agitation. The resulting cell suspension was passed through a 100-μm cell strainer to remove undigested debris, washed twice in PBS containing 2% fetal bovine serum (FBS), and counted. Matched peripheral-blood mononuclear cells (PBMCs) were isolated from the same patients by density-gradient centrifugation over Ficoll-Paque PLUS (Cytiva) according to the manufacturer’s protocol. Both thrombus-derived single cells and PBMCs were cryopreserved in 90% FBS/10% DMSO and stored in liquid nitrogen until CyTOF analysis.

#### Mass cytometry antibody staining and data acquisition

Cryopreserved thrombus-derived cells and PBMCs were thawed separately in pre-warmed RPMI-1640 medium supplemented with 10% FBS, washed, and counted. Cells were incubated with 25 μM Cell-ID Cisplatin-198Pt (Standard BioTools) for 5 min at room temperature for live/dead discrimination, followed by Fc-receptor blocking using Human TruStain FcX (BioLegend) according to the manufacturer’s instructions. Cells were subsequently stained with the metal-conjugated antibody panel (35 markers; full panel listed in the key resources table and visualized in Figure S1) targeting lineage, differentiation, activation, immune-checkpoint, cytokine receptor, transcription-factor, and functional readouts. For intracellular and intranuclear staining (FOXP3, T-bet, Eomes, Ki-67, TNF-α, IFN-γ, Perforin), cells were fixed and permeabilized using the FOXP3/Transcription Factor Staining Buffer Set prior to antibody incubation. After surface and intracellular staining, cells were labeled overnight at 4 °C with Cell-ID Intercalator-Ir (Standard BioTools) in Maxpar Fix and Perm buffer for DNA identification. Before acquisition, cells were washed and resuspended in Maxpar Cell Acquisition Solution containing 10% EQ Four Element Calibration Beads (Standard BioTools), filtered through a 40-μm cell strainer, and adjusted to a final concentration of 5 × 10^5^ cells/mL. Samples were acquired on a Helios mass cytometer (Standard BioTools, formerly Fluidigm). Raw FCS files were normalized using the EQ Passport bead-based normalization method according to the manufacturer’s recommendations, and CyTOF files were further cleaned using the FlowJo CyTOF plugin, followed by manual gating to exclude normalization beads, dead cells, doublets, and debris prior to downstream analyses.

#### Dimensionality reduction and clustering

CD45^+^ events from thrombi and paired PBMCs were exported and analyzed using an R-based semi-automated pipeline (R v 4.0.0). Two visualization algorithms were used. For the CyTOF data, t-distributed stochastic neighbor embedding (t-SNE) was used because t-SNE preserves local marker-defined cluster structure well at the resolution of the immune subsets profiled, and Phenograph was used for unsupervised graph-based clustering. For the integrated scRNA-seq atlas drawn from the BioTuring Talk2Data v5 platform, Uniform Manifold Approximation and Projection (UMAP) was used because UMAP preserves both local and global structure and is the standard embedding used by that platform. For Figures 2C, 2D, 2G, 2H, and 4, the same CyTOF data were re-embedded by UMAP for visual consistency with the scRNA-seq panels. Major immune-lineage assignments were concordant between the t-SNE and UMAP embeddings of the CyTOF data. Single-cell data were analyzed in Python (v 3.13.3) for downstream visualization of the integrated scRNA-seq atlas.

### Quantification and statistical analysis

#### Compositional analyses and group comparisons

All analyses are based on relative cell-type proportions of the live CD45^+^ (or, for the non-immune compartment analysis, CD45^−^) compartment in each specimen, rather than on absolute cell counts. Differential cluster abundance between non-cancer DVT and CAT was assessed by fold change with paired two-tailed Student’s t-tests at the per-cluster level, with Benjamini-Hochberg false discovery rate (BH-FDR) correction applied across all clusters within each comparison. Because single-cell compositional data are inherently relative, an apparent change in any one population can in principle reflect a change in the abundance of another population within the same compartment, and the language used throughout the manuscript (“relative increase”, “relative reduction”, “relative enrichment”) reflects this caveat. Cancer-type-stratified panels (Figures 4 and 5) are presented as descriptive comparisons of cluster frequencies given the limited per-cancer-type sample sizes (*n* = 1 for thyroid, liver, and gastric CAT; *n* = 4 for lung CAT) and are not interpreted as inferential between-cancer comparisons.

#### Exploratory linear regression framework linking clinical covariates to immune-cell proportions

The exploratory regression analyses linking routine hematology and biochemistry to immune-cell proportions (Tables S3, S4, S5A, and S5B) were performed on the subset of 10 patients (3 non-cancer DVT and 7 CAT) for whom both CyTOF profiling and patient-level routine biochemistry were available. Patients 2 and 11 (non-cancer DVT) lacked routine biochemistry and were excluded entirely; Patient 15 (lung CAT) had biochemistry available except for sodium and chloride, and therefore contributed to all regressions involving the other six predictors but was excluded from sodium- and chloride-related regressions, reducing the effective sample size for those two predictors only from 10 to 9 patients per tissue (and from 20 to 18 paired observations in the combined model). These regression analyses are explicitly exploratory and are intended to generate hypotheses rather than to support causal inference.

Cell-type proportions were log-transformed as ln(1 + value), and laboratory variables were standardized to z-scores [(x − mean)/SD] so that regression coefficients reflect a one-standard-deviation change in the predictor. Two complementary frameworks were used.

First, tissue-stratified univariable ordinary least-squares (OLS) regressions (Tables S4 and S5A) were fitted separately on PBMC and thrombus samples. For each immune subset and each clinical predictor, the model was:

$$\ln\left(1+\text{Cell}ᵢ\right)=\beta_{0}+\beta_{1}\;\cdot \;Z\left(\text{Predictor}ᵢ\right)+\beta_{2}\;\cdot \;\text{Type}ᵢ+\varepsilon ᵢ$$

where Cellᵢ is the proportion of the immune subset for participant i, Z(Predictorᵢ) is the z-scored clinical predictor, Typeᵢ is a binary covariate adjusting for cancer status (0 = cancer, 1 = non-cancer), and εᵢ are independent residuals assumed to be normally distributed with constant variance. The primary coefficient of interest, β_1_, represents the expected change in log-transformed cell abundance for a one-standard-deviation rise in the predictor, independent of cancer status.

Second, a combined paired-sample regression model integrating PBMC and thrombus observations from the same patients (Table S5B) was fitted as:

$$\ln\left(1+\text{Cell}ᵢⱼ\right)=\beta_{0}+\beta_{1}\;\cdot \;Z\left(\text{Predictor}ᵢ\right)+\beta_{2}\cdot \;\text{Type}ᵢ+\beta_{3}\cdot \;\text{Tissue}ⱼ+\varepsilon ᵢⱼ$$

where j indexes tissue (PBMC vs. thrombus) and Tissueⱼ is a binary indicator for thrombus (0 = PBMC, 1 = thrombus). This combined paired-sample model takes advantage of the within-patient pairing of PBMC and thrombus specimens to gain statistical power for predictor effects acting consistently across compartments.

Precision was quantified using two-sided Wald 95% confidence intervals. To account for multiple testing, the Benjamini-Hochberg false discovery rate (BH-FDR) procedure was applied within each immune-cell subset (per-outcome family of eight predictors), reflecting the independent biological hypotheses tested for each subset. Both raw *p*-values and per-outcome BH-FDR-adjusted q-values are reported in Tables S3, S4, S5A, and S5B. Associations with FDR q < 0.05 were considered statistically robust; associations with raw *p* < 0.05 but FDR q ≥ 0.05 are reported as nominal trends warranting validation in larger cohorts. All regression analyses were performed in R (v 4.3.2).

## Supplemental information

Document S1. Figures S1–S6 and Tables S1–S3 and S6

Table S4. Full statistics for the thrombus-compartment linear models, listing each immune-cell outcome and clinical predictor with its coefficient, raw *p* value, 95% confidence interval, per-outcome Benjamini-Hochberg FDR value, and sample size

Table S5. Full statistics for the exploratory regression models linking routine clinical indices to immune-cell proportions, listing each outcome and predictor with its coefficient, raw *p* value, 95% confidence interval, per-outcome Benjamini-Hochberg FDR value, and sample size (A) Univariable models fitted in the PBMC compartment. (B) Combined paired-sample model pooling matched PBMC and thrombus specimens from the same patients.
