# Supplementary material for: Single-cell immune profiling of blood and thrombus in cancer-associated thrombosis
Source: iScience. 2026 Aug 20;29(9):117151. doi: 10.1016/j.isci.2026.117151 (PMC13524497; doi:10.1016/j.isci.2026.117151)
Supplement: Supplementary file 1 [file mmc1.pdf]

iScience, Volume *29*

Supplemental information

Single-cell immune profiling of blood and thrombus in cancer-associated thrombosis

Linlin Zhou, Yin Xia, Bingkun Chen, Xiaowen Lin, Hung-Chih Yang, and Chen-Wei Yu

Immune context	
CD45	Pan immune cells
CD19	B cells
CD3	T cells
CD4	CD4 T cells
CD8a	CD8 T cells
CD11b	Myeloid cell
CD56	NK cells
CD16	Granulocyte
FOXP3	Treg master regulator
CD45RO	Memory t cells
CD45RA	Naïve t cells
CD14	Monocyte
CD15	Granulocyte
CD68	Macrophage

Immunomodulatory molecules	
PD-L1	Coinhibitory ligand
HLA-DR	Ag presentation
PD-1	Coinhibitory R.
LAG-3	Coinhibitory R.
CTLA-4	Coinhibitory R.
4-1BB	Costimulatory R.
CD86	Coinhibitory ligand
Tbet	Regulate Th1 cytokine
CD27	T cell activation
CD160	HVEM ligand
Eomes	Differentiation
TNFa	Proinflammatory
IFNg	Cytotoxicity
Perforin	Cytotoxicity

Cytokine/chemokine R	
IL-6	Inflammation/B cells
IL-17A	Proinflammatory cytokine
CCR7	Chemokine R7
CD183	CXCR3

Function	
Ki-67	Cell division

Adhesion molecules	
CD31	Endothelial cells

Ectoenzyme	
CD38	cADP ribose hydrolase

Figure S1. Composition of the CyTOF antibody panel used to profile thrombus and blood immune cells.

The panel combines lineage, differentiation, activation, and functional markers in six colour-coded categories. The "Immune context" block (peach) lists canonical identifiers for major leukocyte lineages including CD45 for all haematopoietic cells; CD19 for B cells; CD3, CD4 and CD8a for T-cell subsets; CD56 for NK cells; CD11b, CD14, CD15, CD16 and CD68 for myeloid and granulocyte populations and together with memory (CD45RO), naïve (CD45RA) and regulatory T-cell (FOXP3) markers. Immunoregulatory checkpoints and effector molecules appear in the green block, encompassing coinhibitory receptors and ligands (PD-1, PD-L1, CTLA-4, LAG-3, CD160), costimulatory or activation markers (4-1BB, CD27, CD86), the antigen-presentation molecule HLA-DR, lineage-defining transcription factors (T-bet, Eomes), and inflammatory or cytotoxic mediators (TNF-α, IFN-γ, perforin). Cytokine and chemokine-receptor markers (magenta) include IL-6R, IL-17RA, CCR7, and CXCR3, while CD38 (slate blue) captures ectoenzyme activity linked to cellular metabolism. Functional state is further gauged by Ki-67 (cyan) for proliferation and by the endothelial adhesion molecule CD31 (gold) for vascular interaction.

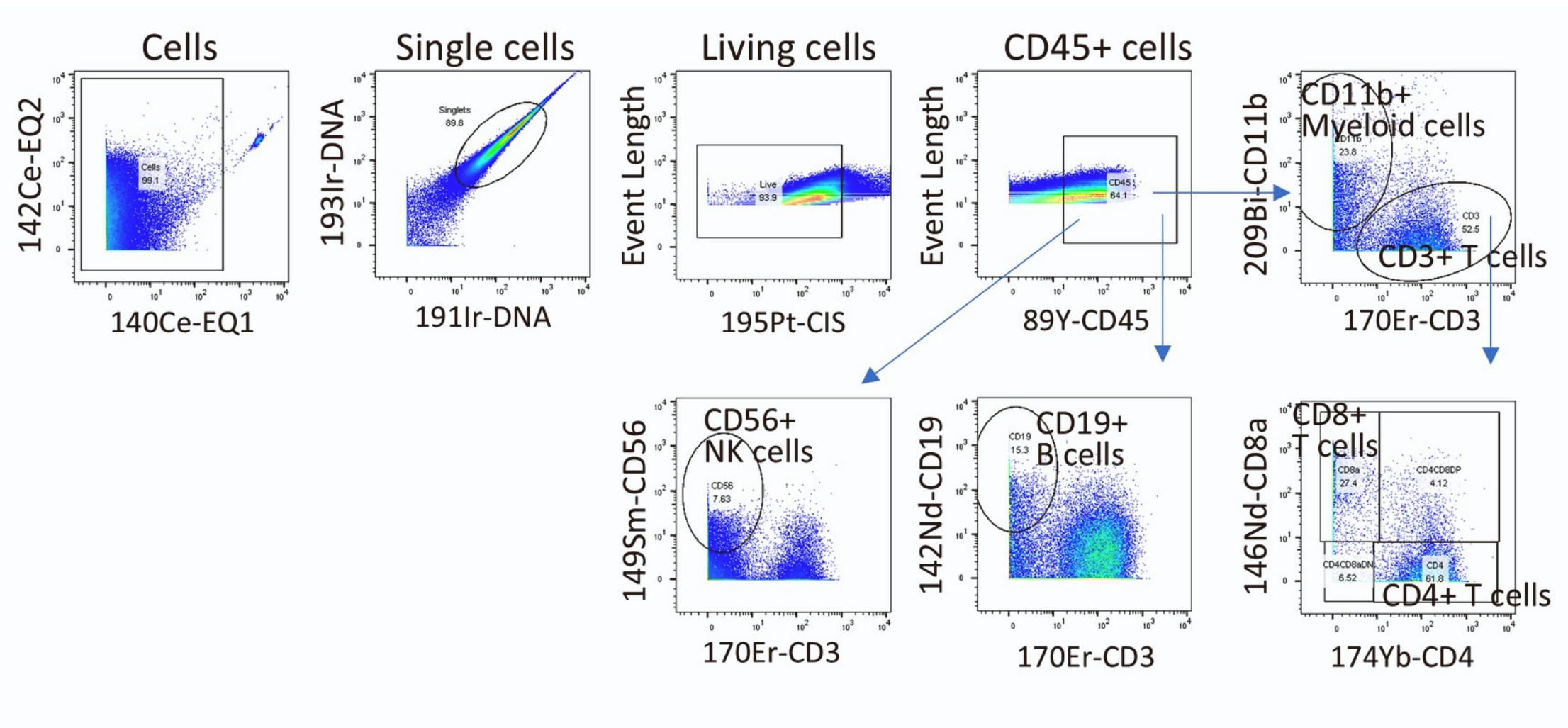

Figure S2. CyTOF gating hierarchy used to resolve major immune lineages in thrombus and peripheral blood.

The figure illustrates the sequential flow-cytometric strategy applied to the 40-parameter CyTOF data. Cellular events were first separated from calibration beads in the 140Ce-EQ1 versus 142Ce-EQ2 plot; singlets were then selected by their tight diagonal signal on the 191Ir-DNA versus 193Ir-DNA bivariate plot, and non-viable events were excluded on the basis of high cisplatin uptake relative to event length. Within the live-cell gate, immune cells were identified by bright 89Y-CD45 staining. From this CD45^{+} compartment, lineage markers were used in two dimensions to distinguish CD11b^{+}CD3^{-} myeloid cells from CD3^{+}CD11b^{-} T cells. The CD3^{-} population was further partitioned into CD56^{+} natural killer cells and CD19^{+} B cells, while CD3^{+} T cells were subdivided into CD8^{+} cytotoxic and CD4^{+} helper subsets. This hierarchical approach yielded mutually exclusive gates for myeloid, NK, B, CD4^{+} T and CD8^{+} T cells, ensuring consistent quantification of each lineage across every thrombus and blood sample analyzed.

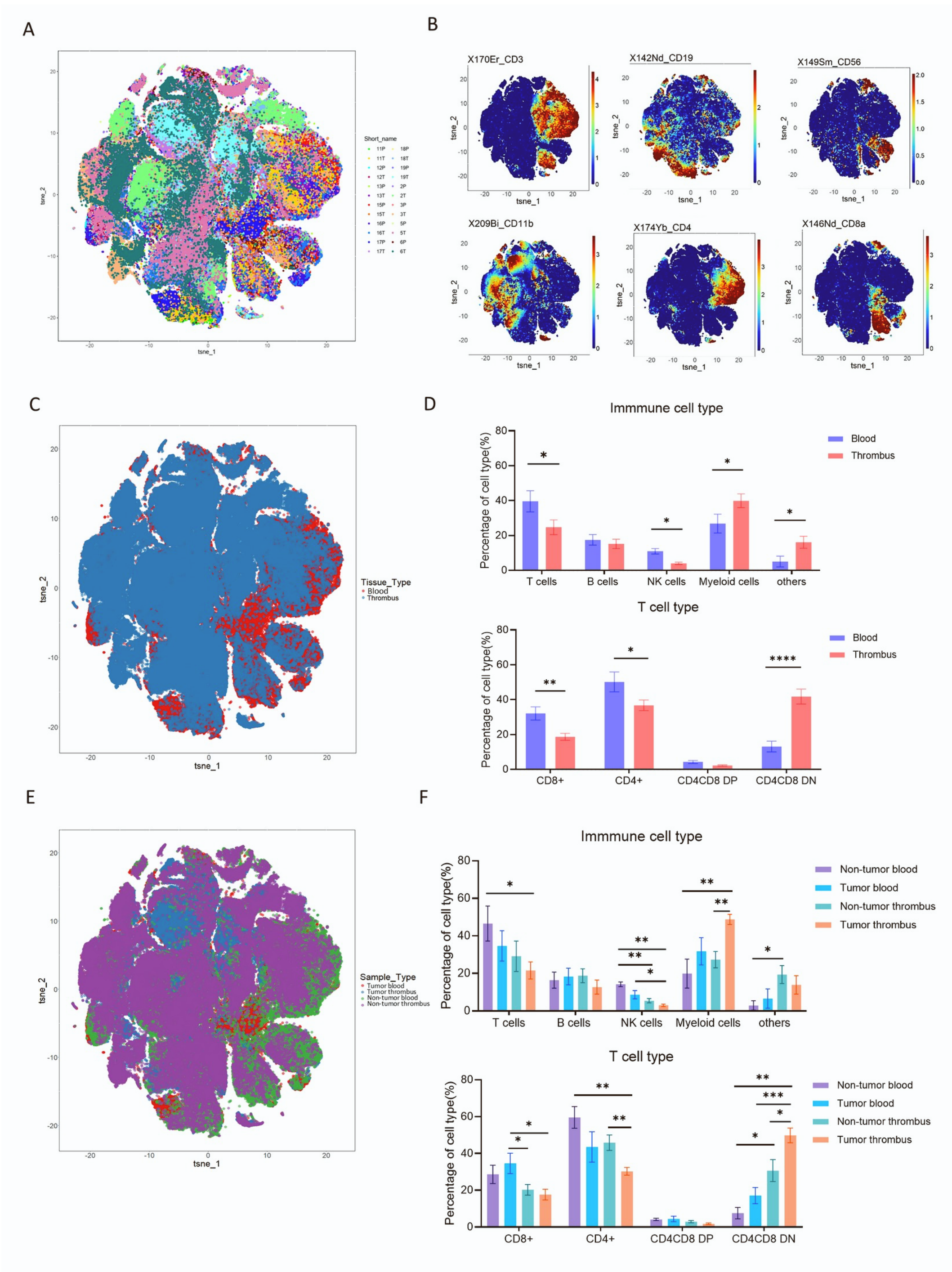

Figure S3. Global distribution of immune cell populations in peripheral blood and thrombus from cancer and non-cancer patients.

A concatenated dataset of 2.4×10^5 $CD45^+$ events (10 000 cells per sample; 24 samples) was analyzed by t-SNE and Phenograph clustering. (**A)** Two-dimensional map colored by unsupervised cluster identity delineates the major immune cells communities present across all specimens. **(B**) Heat overlays of canonical lineage markers including CD3, CD19, CD56, CD11b, CD4 and CD8a confirm annotation of T cells, B cells, NK cells and myeloid cells. (**C)** The same projection colored by compartment distinguishes cells

originating from peripheral blood (red) and thrombus tissue (blue). (**D)** Bar charts (mean ± sd.) summaries the relative abundance of broad lineages (T, B, NK, myeloid, others; upper panel) and T-cell subsets ($CD8^+$, $CD4^+$, $CD4^+CD8^+$ double-positive, $CD4^-CD8^-$ double-negative; lower panel) in blood versus thrombus; significance assessed by two-tailed unpaired Student's t-test. **(E)** Four-way comparison of the t-SNE map colored by sample category including non-cancer blood, cancer blood, non-cancer thrombus and cancer-associated thrombus highlighting compartment- and disease-specific clustering. (**F)** Corresponding quantifications of lineage distributions (upper) and T-cell subset frequencies (lower) across the four groups (mean ± sd.); differences evaluated by one-way ANOVA with Tukey post-hoc test. *$p < 0.05$; **$p < 0.01$ and ***$p < 0.001$.

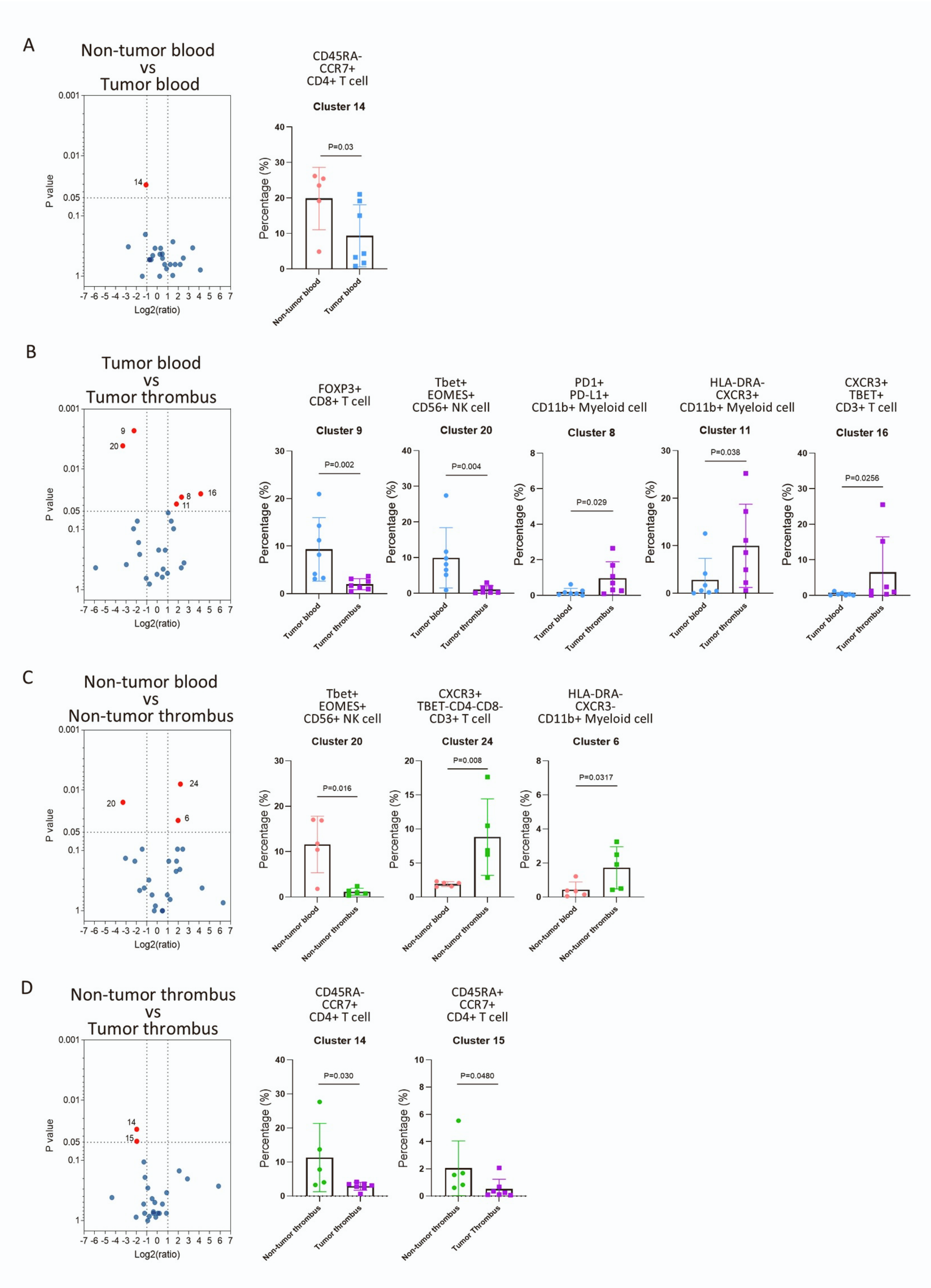

Figure S4. Differential representation of single-cell immune clusters across blood and thrombus compartments in cancer and non-cancer patients.

For each comparison, the relative abundance of Phenograph-defined CyTOF clusters was quantified in paired specimens and visualized as a volcano plot (left; x-axis, $\log_2$ fold change; y-axis, $-\log_{10}$ P value; two-tailed unpaired Student's t-test). Box-and-whisker plots (right; mean ± sd) depict the percentages of the highlighted clusters in each sample group. (**A**) Non-cancer peripheral blood versus cancer peripheral blood; a

selective reduction of CD45RA-CCR7^{+} central-memory CD4^{+} T cells (cluster 14) is observed in non-cancer patient blood. (**B)** Cancer peripheral blood versus cancer-associated thrombus; thrombi are downregulated for FOXP3^{+} CD8^{+} T cells with a regulatory-like phenotype (cluster 9) and T-bet^{+}EOMES^{+} NK cells (cluster 20), but upregulated for PD-1^{+}PD-L1^{+} CD11b^{+} myeloid cells (cluster 8), HLA-DRA-CXCR3^{+} CD11b^{+} myeloid cells (cluster 11), and CXCR3^{+}T-bet^{+} CD3^{+} T cells (cluster 16). (**C)** Non-cancer peripheral blood versus non-cancer thrombus; thrombi show accumulation of CXCR3^{+}T-bet^{-} double-negative (CD4^{-}CD8^{-}) T cells (cluster 24) and HLA-DRA^{-}CXCR3^{-} CD11b^{+} myeloid cells (cluster 6), whereas T-bet^{+}EOMES^{+} NK cells (cluster 20) are more abundant in blood. (**D)** Non-cancer versus cancer-associated thrombus; tumor thrombi display a pronounced loss of naive/central-memory CD4^{+} T-cell subsets (CD45RA^{+}/-CCR7^{+}; clusters 14 and 15).

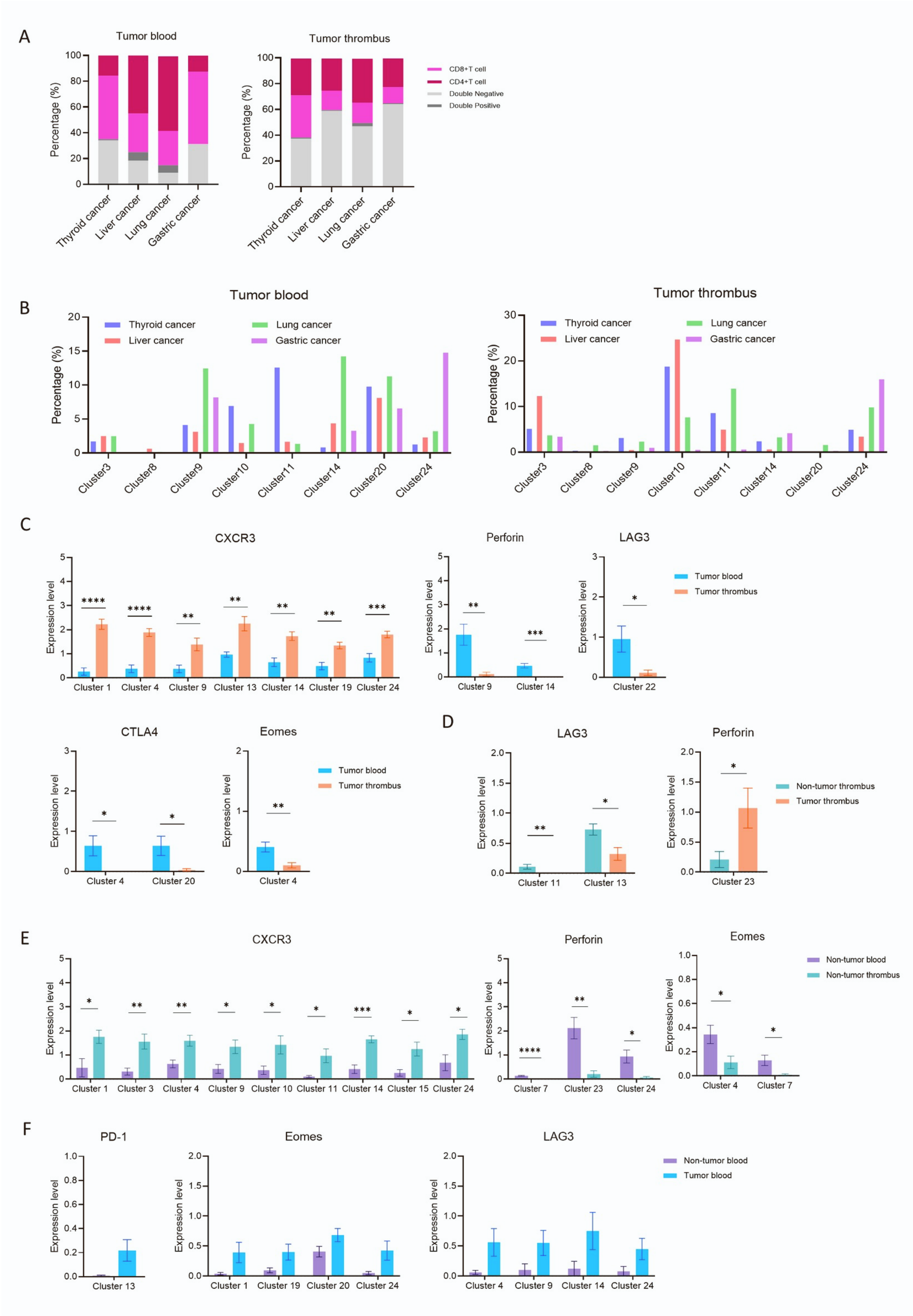

Figure S5. Cancer-type-specific T-cell composition and differential expression of functional immune markers across blood and thrombus compartments.

(A) Stacked columns illustrate the distribution of CD8$^+$, CD4$^+$, double-positive (CD4$^+$CD8$^+$), and double-negative (CD4$^-$CD8$^-$) T-cell subsets within the total CD3$^+$ compartment for four tumor entities, including thyroid, liver, lung, and gastric, shown separately for circulating blood (left) and matched cancer-associated thrombus (right).

(B) Frequencies of key Phenograph clusters that differed between cancers are plotted for tumor blood (left) and tumor thrombus (right); bar colors correspond to cancer type (thyroid, liver, lung, gastric), emphasizing tumor-specific immune signatures. **(C)** The expression levels of selected functional molecules in tumor blood versus tumor thrombus. Chemokine receptor CXCR3 is upregulated in multiple clusters (1, 4, 9, 13, 14, 19, 24) within thrombi, whereas perforin, LAG-3, CTLA4, and EOMES show opposing patterns. **(D)** Comparison of non-cancer and cancer thrombi reveals significantly increased LAG-3 (clusters 13) and perforin (cluster 23) in the malignant setting. **(E)** In non-cancer patients, thrombus tissues display higher CXCR3 (multiple clusters), lower perforin (clusters 7, 23, 24), and lower EOMES (clusters 4, 7) relative to autologous blood. **(F)** Contrasting blood from cancer and non-cancer patients shows enhanced PD-1 (cluster 13) and LAG-3 (clusters 4, 9, 12, 24) together with a modest rise in EOMES (clusters 1, 19, 20) in malignancy, consistent with systemic T-cell exhaustion. Statistical significance in panels C–F was assessed by two-tailed unpaired Student's t-test; $^*p < 0.05$, $^{**}p < 0.01$, and $^{***}p < 0.001$.

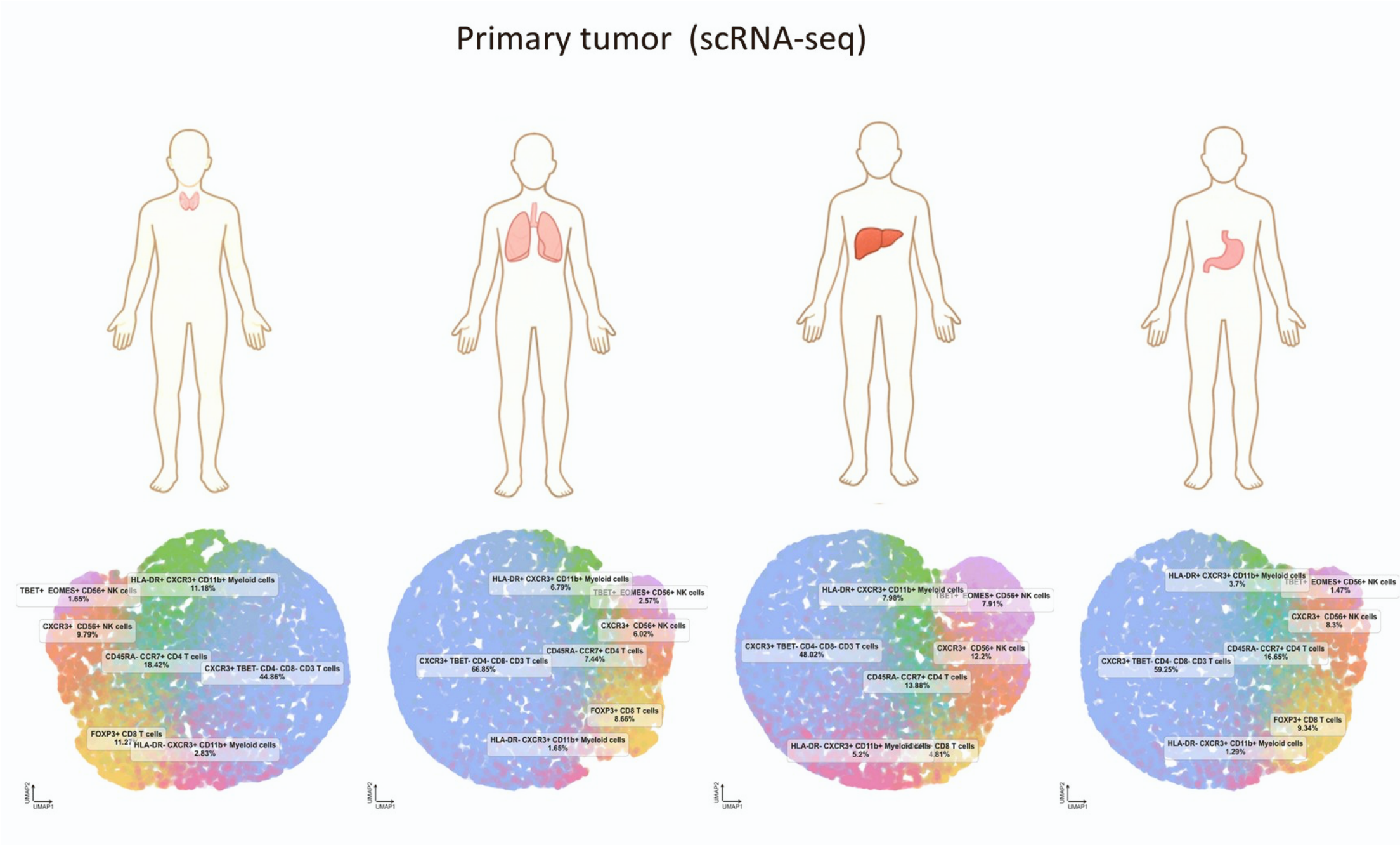

Figure S6. Distribution of thrombus-associated immune phenotypes within primary tumors revealed by single-cell RNA-seq.

Publicly available single-cell RNA-seq datasets for thyroid, lung, liver, and stomach cancers were individually integrated and visualized by UMAP. Each point represents a single immune cell, colored by transcriptionally defined cluster and annotated with its dominant marker profile to allow direct comparison with the CyTOF clusters identified in thrombus tissue. Key phenotypes previously enriched in cancer-associated thrombi, including CXCR3-high T-bet^{+} CD4^{-}CD8^{-} double-negative T cells, PD-1^{+}PD-L1^{+} CD11b^{+} myeloid cells, CXCR3-high CD56^{+} NK cells, and FOXP3^{+}CD8^{+} T cells with a regulatory-like phenotype, are also detectable in the corresponding primary tumors, albeit with tumor-type-specific prevalence.

Table S1. Per-patient clinical and platform availability information for the CyTOF cohort.

Patient ID	Sample IDs (PBMC / Thrombus)	Group	CAT cancer type	CyTOF (PBMC)	CyTOF (Thrombus)	Hematology / Biochemistry
2	2P / 2T	Non-cancer DVT	—	Yes	Yes	No
3	3P / 3T	Non-cancer DVT	—	Yes	Yes	Yes
5	5P / 5T	Non-cancer DVT	—	Yes	Yes	Yes
11	11P / 11T	Non-cancer DVT	—	Yes	Yes	No
13	13P / 13T	Non-cancer DVT	—	Yes	Yes	Yes
6	6P / 6T	CAT	Thyroid	Yes	Yes	Yes
12	12P / 12T	CAT	Liver	Yes	Yes	Yes
15	15P / 15T	CAT	Lung	Yes	Yes	Partial†
16	16P / 16T	CAT	Lung	Yes	Yes	Yes
17	17P / 17T	CAT	Lung	Yes	Yes	Yes
18	18P / 18T	CAT	Gastric	Yes	Yes	Yes
19	19P / 19T	CAT	Lung	Yes	Yes	Yes
Total Non-cancer DVT (n = 5):				5	5	3
Total CAT (n = 7):				7	7	6 + 1†
Total CyTOF cohort (n = 12):				12	12	9 + 1† + 2 missing

† *Patient 15 (lung CAT): hematology and biochemistry available except for sodium (Na+) and chloride (Cl-), which were missing from the clinical record. This patient was therefore included in regression analyses for the six other predictors (hemoglobin, neutrophil count, RBC count, thrombocyte count, monocyte count, WBC count) but was excluded from regressions involving Na+ or Cl-, reducing the effective sample size for those two specific predictors from n = 10 to n = 9 patients per tissue (and from n = 20 to n = 18 paired observations in Table S5B).*

Table S2. Comparison of clinical and laboratory parameters between tumor and non-tumor patients with DVT.

Factors	Tumor patients (median (95% CI); n=13)	Non tumor patients (median (95% CI); n=12)	Statistics	p value
Age	66.0 (62.0-70.0)	71.0 (67.0-74.0)	Mann-Whitney U	0.091
BUN	5.0 (4.0-9.0)	4.6 (4.1-5.2)	Mann-Whitney U	0.121
Creatinine	70.2 (57.6-102.8)	71.2 (63.9-79.6)	Mann-Whitney U	0.775
ALT	16.4 (6.0-22.7)	22.7 (12.7-43.4)	Mann-Whitney U	0.339
AST	20.5 (12.1-30.0)	26.2 (16.0-47.0)	Mann-Whitney U	0.178
Total Serum Protein	62.1 (60.5-75.8)	67.1 (63.7-79.3)	t test	0.157
Albumin	33.7 (30.4-42.6)	35.0 (33.0-43.0)	t test	0.531
WBC	7.1 (6.2-8.8)	7.0 (6.1-8.6)	Mann-Whitney U	0.843
Hemoglobin	99.0 (78.0-118.0)	118.0 (104.0-126.0)	Mann-Whitney U	0.042
Platelet Count	186.0 (134.0-199.0)	212.0 (177.0-260.0)	t test	0.325
AFP	2.0 (2.0-2.5)	2.0 (1.8-2.9)	Mann-Whitney U	0.935
CEA	3.7 (2.0-17.0)	2.0 (1.7-3.7)	Mann-Whitney U	0.049
Na^+	135.1 (130.7-139.6; n = 12)	139.3 (138.6-141.2; n = 6)	Mann-Whitney U	0.125

Cl^-	99.3 (95.5-103.2; n = 12)	103.7 (102.9-104.7; n = 6)	Mann-Whitney U	0.042

Blood urea nitrogen (BUN; mmol/L), creatinine (umol/L), alanine aminotransferase (ALT; U/L), aspartate aminotransferase (AST; U/L), total serum protein (g/L), albumin (g/L), white blood cell count (WBC; *10^9/L), hemoglobin (g/L), platelet count (*10^9/L), alpha-fetoprotein (AFP; ug/L), Carcinoembryonic Antigen (CEA; ug/L), Na^+ (sodium; mmol/L) and Cl^- (chloride; mmol/L). Na^+ and Cl^- measurements were not performed on all patients in the broader hematology cohort: of the 13 CAT patients, 12 had Na^+/Cl^- measured (Patient 15 lacked these values, as noted in Table S1 footnote), and of the 12 non-cancer DVT patients, 6 had Na^+/Cl^- measured.

Table S3. Hemoglobin (Hb) Associated Effect Sizes for Key Immunoregulatory T-Cell Subsets in Thrombus Versus PBMC

Immune subset	Dataset	β (log-scale)	95 % CI	p value	BH-FDR q (per-outcome)
$FOXP3^{+}$ $CD8^{+}$ T cells	Thrombus	0.427	0.172-0.681	0.005	0.043
$FOXP3^{+}$ $CD8^{+}$ T cells	PBMC	0.673	0.400-0.946	< 0.001	0.005
$CD45RA^{-}$ $CCR7^{+}$ CD4 T cells	Thrombus	0.295	0.021-0.570	0.039	0.155 (n.s.)
$CD45RA^{-}$ $CCR7^{+}$ CD4 T cells	PBMC	0.560	-0.046-1.165	0.065	(n.s.)

Table S6. Sample numbers for each experimental platform.

Method	Sample type	Classification	Sample number (N)
CyTOF	Blood	DVT (Deep venous thrombosis)	5
		CAT (Cancer-associated thromboembolism)	Lung cancer (4)<br>Liver cancer (1)<br>Gastric cancer (1)<br>Thyroid cancer (1)
	Thrombus	DVT	5

Method	Sample type	Classification	Sample number (N)
		CAT	Lung cancer (4)<br>Liver cancer (1)<br>Gastric cancer (1)<br>Thyroid cancer (1)
scRNA-seq<br>(Talk2Data database)	Blood	Normal	1.63×10^7 cells
		Cancer	3.6×10^6 cells
	Primary tumor tissue	Normal	6.85×10^5 cells
		Cancer	Lung cancer (1.9×10^6 cells)<br>Liver cancer (2.14×10^5 cells)<br>Gastric cancer (1.13×10^5 cells)<br>Thyroid cancer (7.18×10^4 cells)
Hematological and biochemical profiles<br>(broader cohort, Table S2)	Blood	DVT	12 patients
		CAT	13 patients
Exploratory regression analyses<br>(Tables S4, S5A, S5B)	Paired blood + thrombus	Non-cancer DVT	3 patients<br>(6 paired observations)
		CAT†	7 patients

Method	Sample type	Classification	Sample number (N)
			(14 paired observations)

CyTOF: mass cytometry; PBMC: peripheral blood mononuclear cells; CAT: cancer-associated thrombosis; DVT: deep vein thrombosis; scRNA-seq: single-cell RNA sequencing.
